# Sirtuins in the phylum Basidiomycota: A role in virulence in *Cryptococcus neoformans*

**DOI:** 10.1038/srep46567

**Published:** 2017-04-21

**Authors:** Samantha D. M. Arras, Jessica L. Chitty, Maha S. I. Wizrah, Paige E. Erpf, Benjamin L. Schulz, Milos Tanurdzic, James A. Fraser

**Affiliations:** 1 Australian Infectious Diseases Research Centre, Queensland, Australia; 2 School of Chemistry & Molecular Biosciences, The University of Queensland, Brisbane, Queensland, Australia; 3 School of Biological Sciences, The University of Queensland, Brisbane, Queensland, Australia

## Abstract

Virulence of *Cryptococcus neoformans* is regulated by a range of transcription factors, and is also influenced by the acquisition of adaptive mutations during infection. Beyond the temporal regulation of virulence factor production by transcription factors and these permanent microevolutionary changes, heritable epigenetic modifications such as histone deacetylation may also play a role during infection. Here we describe the first comprehensive analysis of the sirtuin class of NAD+ dependent histone deacetylases in the phylum Basidiomycota, identifying five sirtuins encoded in the *C. neoformans* genome. Each sirtuin gene was deleted and a wide range of phenotypic tests performed to gain insight into the potential roles they play. Given the pleiotropic nature of sirtuins in other species, it was surprising that only two of the five deletion strains revealed mutant phenotypes *in vitro*. However, cryptic consequences of the loss of each sirtuin were identified through whole cell proteomics, and mouse infections revealed a role in virulence for *SIR2, HST3* and *HST4*. The most intriguing phenotype was the repeated inability to complement mutant phenotypes through the reintroduction of the wild-type gene. These data support the model that regulation of sirtuin activity may be employed to enable a drastic alteration of the epigenetic landscape and virulence of *C. neoformans*.

With the dramatic expansion in the immunocompromised population over the past 30 years, systemic mycoses have become an ever-increasing menace to public health^1^,^2^,^3^. A large number of individuals are now at risk of succumbing to deadly fungal infections, which pose a global concern due to the limited antimycotic treatments available^4^. A leading cause of these infections, particularly in those suffering from AIDS, is the basidiomycete yeast *Cryptococcus neoformans.*

The ability of *C. neoformans* to infect is dependent on a range of virulence factors^5^,^6^. Once within the host, its capacity to grow at 37 °C is essential for survival, but insufficient to enable infection on its own. The fungus also counteracts the immune system *via* several mechanisms, ranging from a capsule that interferes with phagocytosis^7^ to melanin that protects against oxidative killing^5^. Dissemination is facilitated through the action of enzymes such as extracellular proteases and phospholipase B assisting in the invasion of the lung^8^,^9^, and urease promoting entry into the central nervous system^10^,^11^.

Production of the virulence characteristics that contribute to the infection process is regulated by a diverse spectrum of environmental cues, and several transcription factors that mediate these temporal responses are known. Mutants lacking such transcription factors often have complicated, almost counterintuitive phenotypes. For example, strains lacking the iron-responsive transcription factor Cir1 exhibit reduced capsule production and poorer growth at 37 °C, but enhanced production of melanin; the mutant is less virulent^12^. In contrast, deletion of the nitrogen metabolite repression-mediating Gat1 transcription factor results in poorer capsule production, but enhanced melanization at high temperature, ability to grow at temperatures exceeding 40 °C, and slightly enhanced virulence^13^. Strains mutated for the pH response regulator Rim101 also produce less capsule, but are in this case hypervirulent^14^.

In addition to these traditional regulatory mechanisms, the production of virulence factors by *C. neoformans* can be permanently altered *via* other means during the infection process; strains passaged through rabbits or mice can exhibit enhanced virulence in subsequent infections^15^,^16^,^17^, and passage through the human host also produces strains with altered virulence characteristics^18^,^19^,^20^. These kinds of permanent changes have been shown in several instances to be the result of the rapid acquisition of heritable, adaptive mutations during infection, a process we have dubbed a “microevolutionary burst”^21^. The ongoing molecular characterization of microevolutionary changes in this species has revealed mutations that influence processes such as glucose metabolism at human body temperature, melanin production, and dissemination^20^,^22^.

Between the temporal regulation of virulence factor production by transcription factors and the heritable, permanent microevolutionary changes resulting from adaptive mutations, there is a third broad class of regulation of virulence factor production: heritable epigenetic changes. One facet of this response is the remodeling of chromatin through the acetylation of histones, providing broad changes to transcription factor access across the genome for promoters of specific stress response and virulence-related genes. A key complex involved in this process is SAGA, whose components Gcn5 and Ada2 are essential to virulence^23^,^24^. While the importance of histone acetylation *via* the SAGA complex on pathogenesis has been established in *C. neoformans*, the potential role of histone deacetylation in virulence has not.

The best-studied example of epigenetic regulation *via* histone deacetylation in the Fungi is silencing in the model yeast *Saccharomyces cerevisiae*. While haploid *S. cerevisiae* cells are either *MAT***a** or *MAT*α, they all contain the genetic information to be either mating type; two additional, silenced copies of the mating type information exist (*HML* and *HMR*) whose information can be transferred into the *MAT* locus to switch sexual identity. The silencing of *HML* and *HMR* is essential to maintaining either *MAT***a** or *MAT*α cell identity, and is mediated by the Silent Information Regulator Sir2^25^,^26^. Silencing by Sir2 also influences transcription of telomeric genes, contributing to stabilization and protection of the chromosome ends^27^,^28^, and maintenance of the repeated array of rDNA copies, up to half of which are silenced^29^.

*S. cerevisiae* Sir2 is the archetype of the sirtuin class of NAD+-dependent histone deacetylases, and together with Hst1, Hst2, Hst3 and Hst4 represents the sirtuins in this species. These enzymes hydrolyze acetyl-lysine residues on histone tails, leading to more tightly compacted chromatin and creating a transcriptionally silent region^30^,^31^,^32^,^33^,^34^. Found in species ranging from bacteria to eukaryotes^35^, sirtuins bear a characteristic domain containing conserved residues involved in zinc, NAD+ and substrate binding^36^. The function of sirtuins has been linked to processes as diverse as longevity in *Escherichia coli*, transposon silencing in *Oryza sativa* and insulin secretion in humans^37^,^38^,^39^. Importantly, while sirtuins have been characterized in the fungal pathogens *Candida albicans* and *Candida glabrata*, whether they play a role in virulence has not been studied^40^,^41^.

In this study, we report the first broad characterization of the sirtuin class of enzymes performed in a fungus of the phylum Basidiomycota. We identify that *C. neoformans* has five sirtuins encoded within its genome showing varying levels of homology to the sirtuin genes of *S. cerevisiae*. Sequential deletion of the five identified sirtuin genes reveals intriguing but distinct phenotypic differences, indicating that they play diverse roles in *C. neoformans* biology. Focusing on *SIR2,* we employed ChIP-seq to identify the genomic locations it associates with in the *C. neoformans* genome, and revealed that loss of this gene results in decreased virulence. *HST3* and *HST4* are also required for virulence. Surprisingly, none of our identified sirtuin mutant phenotypes could be complemented after the reintroduction of the wild-type gene. We hypothesize that sirtuin inactivation and subsequent reactivation leads to a drastic alteration in the epigenetic information that is encoded by the sirtuins in this important human fungal pathogen.

## Results

### A search for sirtuins in *C. neoformans* reveals five candidate family members

**Figure 1:**
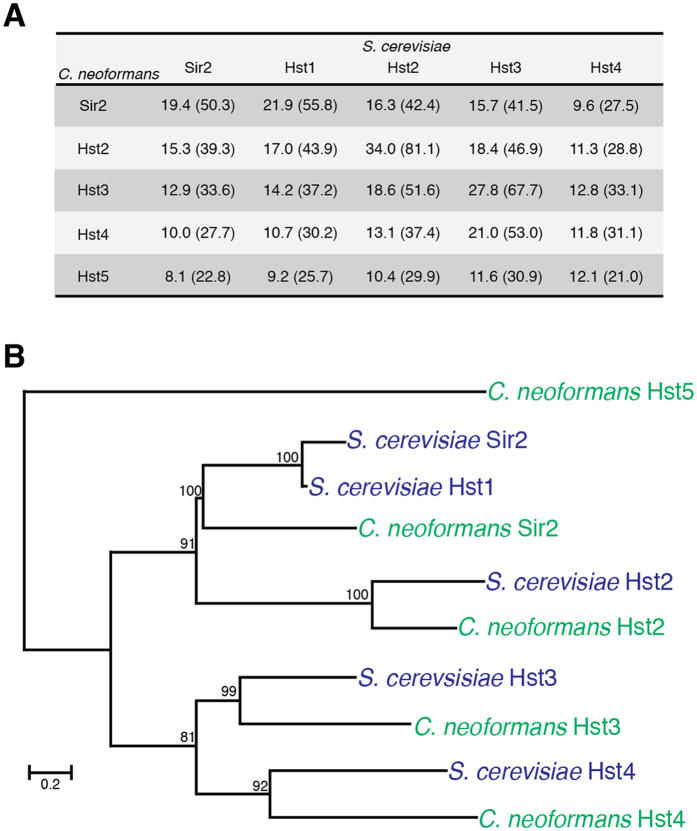
*C. neoformans* has five candidate sirtuins. (**A**) % identity scores (% similarity in parentheses) of predicted *C. neoformans* sirtuins with the *S. cerevisiae* proteins. (**B**) Maximum likelihood phylogenetic tree of the *S. cerevisiae* and *C. neoformans* predicted sirtuins created in MEGA7 with a WAG + G + I model and gamma shape parameters with 500 bootstrap replicates. Scale bar: 0.2 amino acid substitutions per site.

To begin our analysis of the sirtuin class of enzymes, we employed the fungal species in which these enzymes were first identified. Utilizing the five known sirtuins of *S. cerevisiae* (Sir2, Hst1, Hst2, Hst3 and Hst4), reciprocal BLASTp analyses were employed to identify candidate homologous proteins encoded in the genome of the *C. neoformans* type strain H99. Five hits were identified that exhibited both homology to the enzymes from *S. cerevisiae*, and contained a canonical sirtuin domain (Fig. 1, Supplementary Fig. S1). Equivalent results were achieved with OrthoMCL.

Consistent with *S. cerevisiae SIR2* and *HST1* being ohnologs^42^, the best hit for both, based on % protein identity and phylogeny, was locus *CNAG_04866*, which we have dubbed *C. neoformans SIR2* (Fig. 1A and B). *S. cerevisiae* Hst2 showed highest identity and phylogenetic relatedness to the predicted product of *CNAG_07712 (C. neoformans HST2*), and Hst3 to *CNAG_00343 (C. neoformans HST3*). While *S. cerevisiae* Hst4 showed equivalent homology to the products of all of the predicted *C. neoformans* sirtuin ORFs, it phylogenetically clustered with *CNAG_02085 (C. neoformans HST4*) (Fig. 1A and B). The fifth *C. neoformans* gene, *CNAG_03170 (C. neoformans HST5*), showed low identity to all *S. cerevisiae* sirtuins and was phylogenetically distinct; this predicted sirtuin-encoding gene does not have a clear homolog in *S. cerevisiae* (Fig. 1). While the predicted product of *HST5* displayed equally low similarity to the sirtuins from other members of the phylum Ascomycota (*C. albicans, Schizosaccharmoyces pombe, Aspergillus nidulans, Neurospora crassa*; Supplementary Table S1), it did show 36.5% identity (89.4% similarity) to the unstudied predicted sirtuin gene *Hst4*/*UM05239* in *Ustilago maydis*, suggesting this sirtuin may be specific to the phylum Basidiomycota. BLASTp analysis of the *Cryptococcus gattii* WM276 genome revealed all five *C. neoformans* genes were also present in this sister species.

Comparison of the *S. cerevisiae* and *C. neoformans* sirtuins *via* Muscle alignment revealed that beyond the ohnologous nature of *S. cerevisiae* Sir2 and Hst1, the homology that exists falls almost exclusively within the sirtuin domain, which contains the NAD+, substrate and zinc binding residues required for the function of enzymes in this family (Supplementary Fig. S1).

### Deletion of candidate sirtuins from *C. neoformans*

To begin our investigation of the roles of the sirtuin family in *C. neoformans*, we deleted all five candidate genes in *C. neoformans* type strain H99 using biolistic transformation, acquiring at least two independent deletion transformants per sirtuin. These were confirmed by Southern blot. qRT-PCR analysis of the mutants indicated that the siruin genes did not regulate each other, with the expression of the remaining sirtuin genes unchanged in each mutant (Supplementary Fig. S2).

Intriguingly, we were repeatedly unable to obtain stable transformants when ectopically reintroducing the wild-type gene linked to a nourseothricin resistance cassette; the randomly integrated, nourseothricin resistant transformants were universally unstable. In order to achieve reintroduction of the wild-type gene into each of the sirtuin mutants and thereby fulfill the Molecular Koch’s Postulates^43^, we had to develop a new set of molecular tools for use in *C. neoformans* focusing on the complementation process^44^. Through whole genome analyses we identified an intergenic site that we hypothesized would be an ideal location for construct integration (dubbed the Safe Haven), developed a series of vectors designed to target this site, and successfully showed reproducible complementation of non-sirtuin mutants in proof of principle experiments^44^. Using this approach we were able to successfully reintroduce a single wild-type copy of each sirtuin gene into the Safe Haven site in its respective mutant. In each case qRT-PCR was employed to verify that transcript levels of the Safe Haven constructs were statistically equivalent to wild-type (Supplementary Fig. S3).

### *In vitro* mutant phenotypes were detected in only two of the *C. neoformans* sirtuin mutants

**Figure 2:**
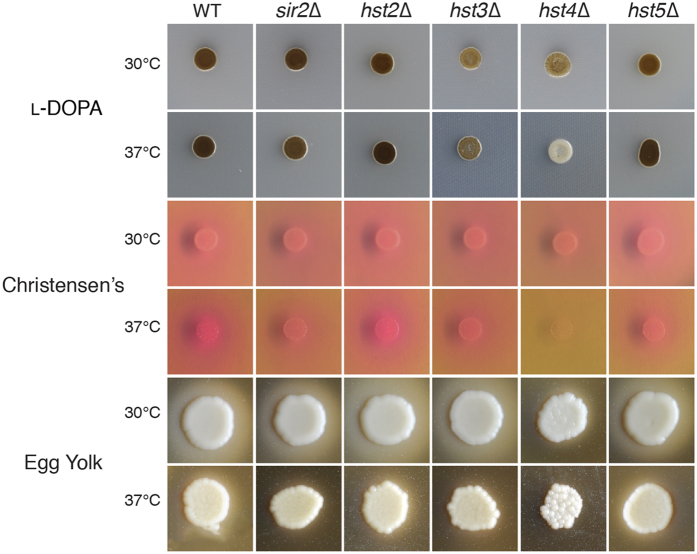
*In vitro* virulence factor production is decreased in only two of the five *C. neoformans* sirtuin mutants. Spot dilution assays on L-DOPA, Christensen’s, and egg yolk agar were used to determine the level of melanin, urease, and phospholipase B production, respectively. Melanized strains appear brown-black, while urease and phospholipase B production appear as halos surrounding the colony. Plates were grown at both 30 and 37 °C.

Performing a battery of phenotypic tests on the sirtuin mutants revealed intriguing phenotypes. Given the pleiotropic nature of some of the sirtuins in other species, we were surprised to observe that none of the deletion strains displayed any growth defects on standard rich (YPD) or minimal (YNB) media at either 30 or 37 °C, in the production of protease or capsule, or the ability to mate in a unilateral cross (Supplementary Fig. S4). The *sir2∆, hst2∆* and *hst5∆* strains were also indistinguishable from wild-type in the production of melanin, urease and phospholipase B. However, the *hst3∆* mutant displayed a slight defect in melanin production, while the *hst4∆* mutant exhibited reduced melanin, urease and phospholipase B production at 30 °C, with a complete absence at 37 °C (Fig. 2). Unexpectedly, none of these phenotypes were complemented in the *hst3∆* or *hst4∆* strains in which the wild-type gene had been reintroduced in single copy at the Safe Haven (Supplementary Fig. S5), a phenomenon we later found to be a characteristic of *C. neoformans* sirtuin mutants.

A range of *in vitro* growth assays, testing a variety of stressors including antifungal resistance, DNA damaging agents, protein synthesis inhibitors, salt stressors, oxidative stressors and cell stressors (Supplementary Fig. S4) also failed to reveal any detectable *in vitro* phenotypes for *sir2∆, hst2∆* and *hst5∆*. Similar to the *in vitro* virulence factor production assays, *hst3*∆ exhibited a number of mutant phenotypes, and *hst4∆* even more. The *hst3∆* mutant displayed increased resistance to fluconazole, and sensitivity to mercaptopurine, fenpropimorph, *t*-butyl hydroperoxide and UV radiation at both 30 and 37 °C. The *hst4*∆ mutant strain was once again the most pleiotropic mutant, with increased sensitivity to fluconazole, itraconazole, UV radiation, NaCl, KCl and cycloheximide at both 30 and 37 °C, and *t*-butyl hydroperoxide, NaNO_2_ and SDS at 37 °C (Supplementary Fig. S6). Again, none of the identified phenotypes were complemented when the wild-type gene was reintroduced into their respective mutants.

### Proteomic analysis of *C. neoformans* sirtuin mutants correlates with the severity of *in vitro* phenotypes

Given the intriguing absence of detectable mutant phenotypes in our panel of *in vitro* tests for three of the sirtuin mutants, we decided to employ a molecular analysis to gain insight into the potential influence of the sirtuins in *C. neoformans*. Given the canonical role of sirtuins is to influence promoter accessibility, both directly and indirectly influencing rates of transcription and thereby protein abundance, we performed SWATH-MS (Sequential Window Acquisition of all Theoretical Mass Spectra) proteomic analysis on wild-type and all five sirtuin mutants to gain insight into whether loss of the *SIR2, HST2* or *HST5* genes had any detectable effect.

**Figure 3:**
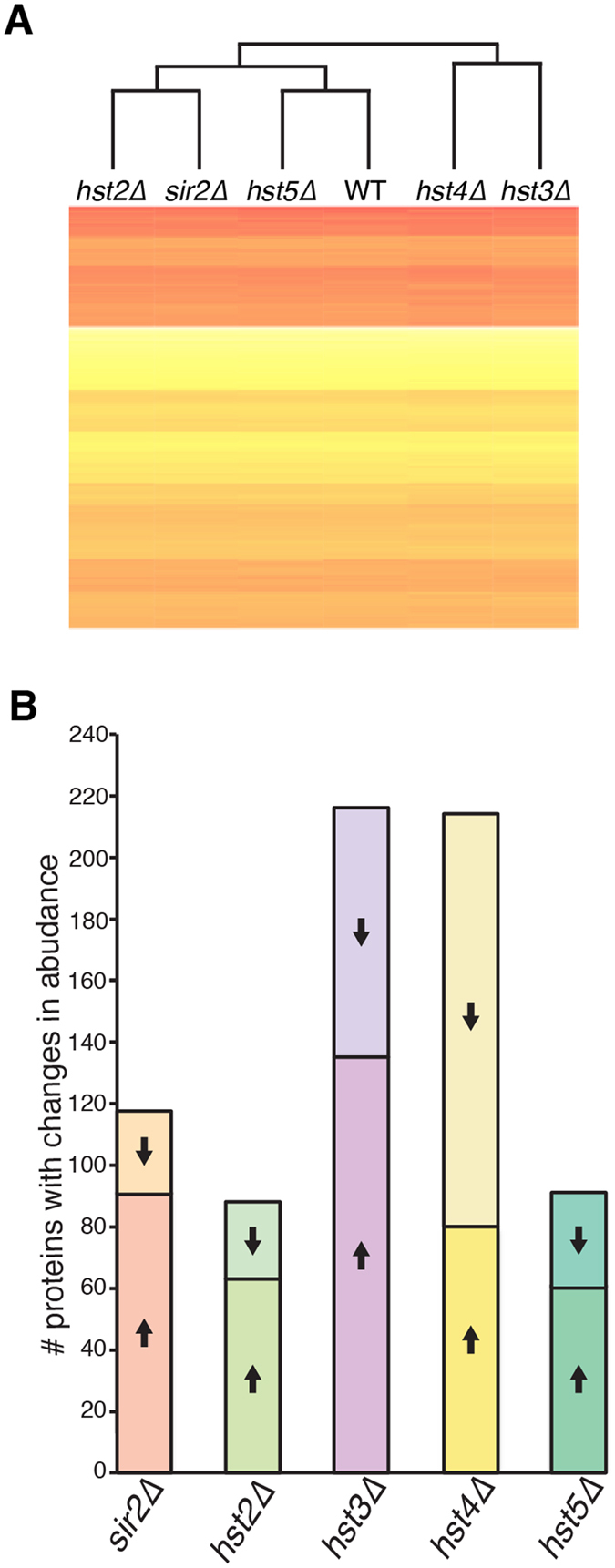
Proteomic analyses reveal a significant number of protein abundance changes in the *C. neoformans* sirtuin mutants. (**A**) Clustered heat map of normalized protein abundances for the wild-type and 5 mutants strains. “Red represents the most abundant proteins, yellow the least abundant”. (**B**) Number of proteins that were identified as significantly increased (up-arrow) or significantly decreased (down arrow) compared with wild type.

Proteomic analysis identified 763 unique proteins from pooled wild-type and mutant samples, and SWATH-MS was used to measure the relative abundance of these in each strain. All sirtuin mutant strains showed a range of significantly different protein abundances compared with wild-type, which in turn were used to generate a clustered heat map of normalized protein abundance (Fig. 3A and B). The proteomic molecular profile was in agreement with the sirtuin deletion phenotypes; the *sir2*∆, *hst2*∆ and *hst5∆* mutants clustered with wild-type and only had 118, 88 and 92 proteins, respectively, with significantly different abundances, consistent with the lack of observed phenotypic changes in these strains (Fig. 2, Supplementary Figs S5 and S6). In stark contrast, the *hst3*∆ and *hst4*∆ mutants formed a separate clade, and had a much greater number of proteins with altered abundance (216 and 214 proteins, respectively); these were also the strains that exhibited multiple mutant phenotypes.

We further analyzed the mass spectrometry data based on Gene Ontology (GO) categories for the biological processes of the proteins that showed difference in abundance between each mutant strain and the wild-type. Based on the GO analysis, the five sirtuins in *C. neoformans* regulate diverse metabolic pathways within the cell, with particular effects of Sir2 on carbohydrate metabolism, Hst2 and Hst4 on nitrogen metabolism, Hst3 on central carbon metabolism and Hst5 on protein biosynthesis (Supplementary Table S2). In short, while our *in vitro* mutant analysis indicated that the sirtuin mutants had either no visible mutant phenotype or a highly complex, pleiotropic phenotype, our proteomic studies highlighted that all five have a significant effect on the function of the cell.

### Identification of Sir2 binding sites in the *C. neoformans* genome

Our genetic and proteomic studies provided evidence that the sirtuins in *C. neoformans* perform very different regulatory roles to their *S. cerevisiae* counterparts. *S. cerevisiae sir2∆* displays numerous easily detectable phenotypes while *S. cerevisiae hst4∆* exhibits few; in contrast, *C. neoformans sir2∆* displayed no detectable mutant phenotypes, while *C. neoformans hst4∆* is highly pleiotropic. To gain insight into this phenomenon, we investigated the differences in DNA association between *S. cerevisiae* Sir2, the sirtuin for which the most extensive ChIP-seq data exists, and its *C. neoformans* homolog.

In *S. cerevisiae*, the function of Sir2 has been extensively studied, and the phenotypes of the mutant can be linked to the regions of the genome the protein associates with. To determine if the genomic targets of *C. neoformans* Sir2 are similar, we HA-tagged *SIR2* at its genomic location and performed ChIP-seq, identifying 95 peaks that were consistent across replicates.

The original function discovered for *S. cerevisiae* Sir2 was its key role as a silencer of the small *HML* and *HMR* loci in the brewing yeast bipolar mating-type system. However, the *C. neoformans* bipolar mating-type system is strikingly different; *MAT* is very large (over 100 kb) and the genome lacks silent cassettes^45^. Given no effect on mating had been observed in the *sir2∆* mutant, and given the very different mating-type system, it was unsurprising that Sir2 did not associate with *C. neoformans MAT*. The closest binding sites were over 50 kb away from either end of the locus, consistent with Sir2 not performing an important function in regulating sexual identity.

A second role attributed to *S. cerevisiae* Sir2 is in influencing transcription of telomeric genes, contributing to stabilization and protection of the chromosome ends^27^,^28^. *C. neoformans* telomeres are very different to those seen in brewing yeast as they are not gene-poor, and there has been no reported evidence of silencing^46^. Consistent with this, Sir2 does not interact with the telomeric regions in this pathogen; the closest binding sites in the ChIP-seq data were a minimum of ~29 kb away from the ends of the chromosomes.

**Figure 4:**
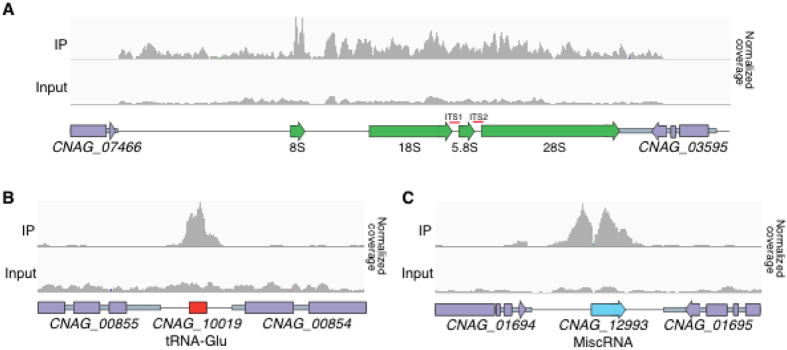
Sir2 association with the *C. neoformans* genome. Representation of CHiP-seq data aligned to the rDNA array (**A**), a tRNA (**B**) and a miscRNA (**C**).

The third function for which *S. cerevisiae* Sir2 is well known is maintenance of the repeated array of rDNA copies^29^. Investigation of the single *C. neoformans* rDNA array on chromosome 2 revealed some similarity between the function of Sir2 in *S. cerevisiae* and *C. neoformans*. However, while *S. cerevisiae* Sir2 association with the rDNA locus is primarily with the ITS regions, in *C. neoformans* Sir2 is instead observed across the length of the rDNA array, accounting for 5 of the 95 identified ChIP-seq peaks (Fig. 4A). No influence on this highly transcribed locus could be detected by qRT-PCR (Supplementary Figure S7).

Of the 90 remaining *C. neoformans* Sir2-HA ChIP-seq peaks, 80 were associated with tRNA genes; further analysis of the tRNA loci revealed that in total, 91% of the 148 tRNA genes in the genome exhibited association with Sir2-HA in one or more ChIP-seq replicates (Fig. 4B). As with the rDNA locus, this association with tRNA genes is also reminiscent of the behavior of *S. cerevisiae* Sir2, which has been identified as displaying affinity for tRNA genes in multiple studies^47^,^48^,^49^. As with the rDNA locus, no influence on these highly transcribed loci could be detected by qRT-PCR (Supplementary Figure S7).

Intriguingly, of the remaining ChIP-seq peaks present in all replicates, 4 were associated with miscRNAs^29^, 3 with genes encoding hypothetical proteins, and 3 with the 3′ regions of convergently transcribed genes with predicted functions (Fig. 4C).

### The loss of *SIR2* results in hypovirulence in a murine model of infection

**Figure 5:**
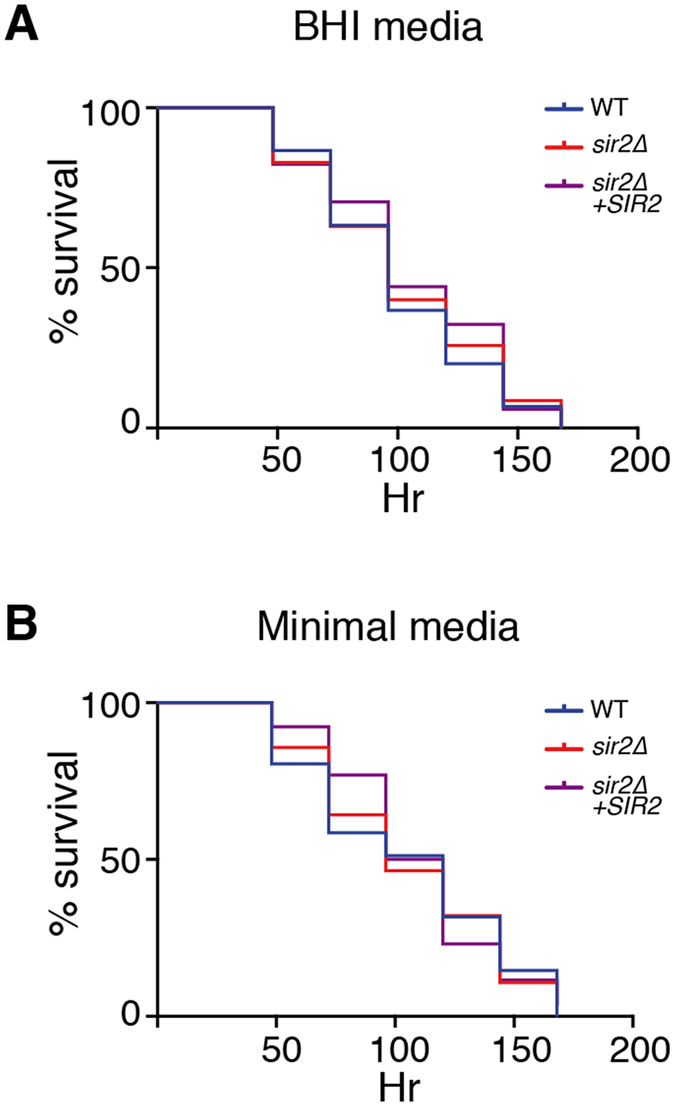
Virulence of sir2Δ in an invertebrate model of infection. Survival of Bristol N2 strain nematodes co-cultured with *C. neoformans* on brain heart infusion or minimal media at 25 °C. *p* =< 0.005.

Although the *sir2*Δ mutant showed no phenotypes on rich or minimal media, or in any of the *in vitro* virulence or stress assays, we speculated that as the infection process poses a greater metabolic stress on the strain, a phenotype might be revealed. We cultured *Caenorhabditis elegans*, a natural predator of *C. neoformans*, on wild type, the *sir2*Δ mutant, and the *sir2*Δ + *SIR2* strain in which *SIR2* has been reintroduced at the Safe Haven site. No significant difference was observed between survival times of the worms on either rich or minimal media (Fig. 5).

**Figure 6:**
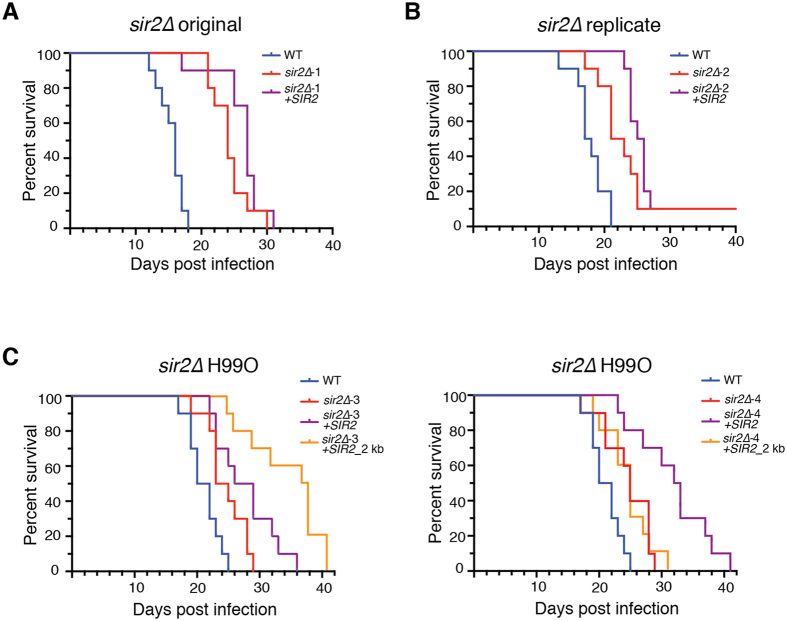
Virulence of sir2Δ in a murine model of infection. Survival of female BALB/c mice infected with 5 × 10^5^ cells *via* nasal inhalation. Four sirtuin strains were subjected to a murine model of infection, with the original *sir2∆* mutant (**A**), a second independent *sir2∆* mutant (**B**), and two *sir2∆* mutants created in the H99O background (**C**) all showing attenuation compared to wild type. Kill curves were considered statistically significant from wild-type when *p* =< 0.05; *sir2∆-*1 (*p* =< 0.001), *sir2∆-*1 + *SIR2 (p* =< 0.001), *sir2∆-*2 (*p* =< 0.001), *sir2∆-*2 + *SIR2 (p* =< 0.001), *sir2∆-*3 (*p* =< 0.001), *sir2∆-*3 + *SIR2 (p* =< 0.001), *sir2∆-*3 (*p* =< 0.001), *sir2∆-*3 + *SIR2_2 kb (p* =< 0.001), *sir2∆-*4 (*p* =< 0.001) and *sir2∆-*4 + *SIR2_2 kb (p* =< 0.001).

We next assessed the requirement of Sir2 for virulence using the murine inhalation model of cryptococcosis. In this model of infection, the *sir2∆* mutant exhibited substantially reduced virulence compared to the wild-type control (*p* =< 0.001) (Fig. 6A). However, perhaps more interesting than the *sir2∆* result was the virulence profile of the *sir2∆* + *SIR2* strain in which the gene was reintroduced at the Safe Haven site. While this strain had a statistically different survival curve than the mutant, wild-type virulence was not restored – reminiscent of our earlier observations that the *hst3∆* and *hst4∆* mutant *in vitro* phenotypes could not be complemented.

Although the lack of complementation of the *sir2∆* mutant phenotype was consistent with our observations of other sirtuin mutants in *C. neoformans*, we further investigated this phenomenon in an effort to determine if there was a simple explanation. Our first model was that the copy of the gene introduced at the Safe Haven site either was not expressed, or was expressed at a level very different from the wild-type locus. Quantitative real-time PCR disproved both of these models; transcription of the Safe Haven *SIR2* was not significantly different from wild-type (Supplementary Fig. S3). We then hypothesized that the *sir2∆* and/or the *sir2∆* + *SIR2* strain had acquired a secondary mutation that gave rise to the unusual virulence result. To address this model we recreated the *sir2∆* mutant (*sir2∆-2*), and created a new *sir2∆* + *SIR2* strain in this background (*sir2∆-2* + *SIR2)*. Once again we employed our murine infection model, and once again we observed hypovirulence in the new *sir2∆* mutant and a lack of complementation of the *sir2∆* hypovirulence (Fig. 6B).

Together, the data we had acquired from the *sir2∆, hst3∆* and *hst4∆* mutants now provided strong evidence that once a sirtuin was removed from *C. neoformans*, restoration of the original function of that enzyme could not be achieved by reintroduction of the wild-type gene. However, we could not discount the possibility that other factors were at play. The H99 genome project revealed that multiple distinct subcultures of this strain with differing virulence existed in the research community; while we were using the lineage that was most commonly employed by other laboratories, we rationalized that a mutation may be present that influences sirtuin function. Alternatively, sirtuin genes in *C. neoformans* may have unusually large, alternative promoters that are only employed during infection. To address these increasingly unlikely scenarios, we independently created two new *sir2∆* mutants (*sir2∆-3* and *sir2∆-4*) in H99O, the same low-virulence subculture used for the genome project. Both mutants were then transformed to introduce *SIR2* at the Safe Haven site with two different clones – one included the original 1 kb of 5′ region (*sir2∆-3* + *SIR2* and *sir2∆-4* + *SIR2*) and the other with 2 kb of 5′ region (*sir2∆-3* + *SIR2_2 kb* and *sir2∆-4* + *SIR2_2 kb*). While each of these new *sir2∆* mutants in the H99O background were hypovirulent, neither had their virulence defect complemented with either the original *SIR2* Safe Haven clone, or the new clone with additional upstream sequence (Fig. 6C). Importantly, in almost all instances these complemented strains exhibited a statistically different virulence than the mutant, indicating that the reintroduced Sir2 was functioning, just not in a way that restored a wild-type phenotype. Together, these data strongly supported our model that loss of sirtuin function in *C. neoformans* cannot be restored simply by reintroducing the wild-type gene.

### The loss of *HST3* and *HST4* also result in hypovirulence in a murine model of infection

**Figure 7:**
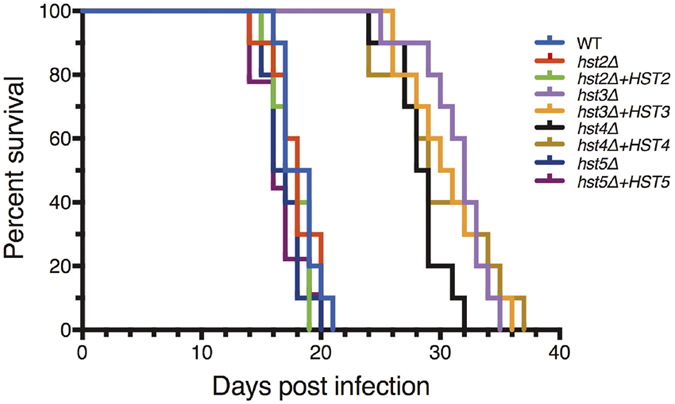
Virulence of hst2Δ, hst3Δ hst4Δ and hst5Δ in a murine model of infection. Survival of female BALB/c mice infected with 5 × 10^5^ cells *via* nasal inhalation. Four sirtuin strains and their derived complemented strains were subjected to a murine model of infection. Kill curves were considered statistically significant from wild-type when *p* =< 0.05; *hst3*Δ (*p* =< 0.001), *hst3*Δ + *HST3 (p* =< 0.001), *hst4*Δ (*p* =< 0.001) and *hst4*Δ + *HST4 (p* =< 0.001).

Given the intriguing inability to complement the virulence defect of the *sir2∆* mutant, we investigated the impact of the remaining sirtuins on the virulence of *C. neoformans* (Fig. 7). Intranasal infection of mice with the *hst2∆, hst3∆, hst4∆* and *hst5∆* mutants along with their derivatives bearing a wild-type allele of the mutated gene at the Safe Haven yielded two key observations. First, the virulence of the mutants again followed the trends observed in our *in vitro* phenotypic assays. The *hst2∆* and *hst5∆* strains, which exhibited few *in vitro* phenotypes, were indistinguishable from wild-type. In contrast, the highly pleiotropic *hst3∆* and *hst4∆* mutants were both attenuated for virulence. Second, reintroduction of the wild-type allele did not restore wild-type virulence in these strains. This result is consistent with the rest of our extensive data indicating that loss of sirtuin function in *C. neoformans* cannot be restored simply by reintroducing the wild-type gene.

## Discussion

While the sirtuin family of proteins have been highly characterized in the model ascomycete yeast *S. cerevisiae*^48^,^50^,^51^ and to a much lesser extent in the pathogenic ascomycete yeasts *C. albicans* and *C. glabrata*^41^,^52^, only one enzyme belonging to this family has been characterized in the phylum Basidiomycota^53^. Here, we describe the identification of five sirtuins in the pathogenic basidiomycete yeast *C. neoformans*. While four of these identified proteins appear to be orthologs of the *S. cerevisiae* sirtuins, we also identified a fifth novel sirtuin (termed Hst5) in *C. neoformans*.

We deleted each of the genes belonging to this family and performed a wide battery of phenotypic tests to gain a broad overview of the potential roles the sirtuins play in *C. neoformans*. Given the pleiotropic nature of some of the sirtuins in other species, we were surprised that only two (*hst3∆* and *hst4∆*) of our five mutant sirtuin strains displayed any detectable defects in the wide variety of growth assays employed. However, we did identify hidden consequences of deleting the other three sirtuin genes (*sir2∆, hst2∆* and *hst5∆*) through whole cell proteomics, revealing that all five sirtuins influence metabolic pathways within the cell.

To further characterize the function of sirtuins in *C. neoformans* we investigated the differences in DNA association between *S. cerevisiae* Sir2, the sirtuin for which the most extensive ChIP-seq data exists, and its *C. neoformans* Sir2 homolog. Given the very different nature of the *MAT* locus and telomeres between the two species, it was unsurprising that we found *C. neoformans* Sir2 does not associate with these genomic features. It does, however, bind to the rDNA array and tRNAs in a fashion similar to that seen in brewers yeast. While highly transcribed regions such as these have been reported to be “hyper-ChIPable”^54^, association with these elements in the genome does not appear to be an artifact of the ChIP-seq process as previous application of this technique investigating phosphate metabolism in *Cryptococcus* did not yield these loci^55^. Furthermore, multiple studies have provided abundant evidence for the functional relevance of Sir2 binding to these highly transcribed loci not only in *S. cerevisiae*, but other species as well^47^,^48^,^49^.

Intriguingly, and perhaps the most fascinating feature of the sirtuins that we observed is the repeated inability to restore wild-type phenotype through the reintroduction of the wild-type gene into the mutant strains. Of the dozens of genes our laboratory has characterized in *C. neoformans*^20^,^22^,^46^,^56^, we always been able to complement a mutant phenotype – but here were three uncomplementable mutants whose only similarity was the family their products belonged to. We therefore hypothesize that the loss of a sirtuin leads to a corresponding loss of otherwise heritable epigenetic information, and after the reintroduction of a wild-type sirtuin into its respective mutant, the epi-genome is not restored to its original state. This model suggests that temporary inactivation of a sirtuin could be a highly effective mechanism for *C. neoformans* to rapidly undergo heritable microevolutionary change without altering its genetic material.

The model that sirtuin inactivation and subsequent reactivation could be employed to enable a drastic re-architecture of the epigenetic landscape of *C. neoformans* is highly complimentary with some key observations already made in this important fungal pathogen. Phenotypic switching in *C. neoformans* has been suggested to be an epigenetically driven process that allows the fungus to change phenotypes without the risk of mutation. Phenotype switch variants have been shown to exhibit enhanced virulence in a murine infection model and are therefore selected in the host environment^57^,^58^. One key observation is that the hypervirulent switch variants appear to have a shortened replicative lifespan^59^. This finding suggests that lifespan is regulated and not fixed, and that phenotypic switching may be epigenetically linked to replicative aging; aging of yeast cells also promotes an increased rate of switching to hypervirulent variants in *C. neoformans*^60^. However, the molecular mechanism of phenotypic switching in *C. neoformans* is unknown. Sir2 has been implicated in aging^61^ and phenotypic switching^41^ in other fungal species, and recent findings by Fries and colleagues^53^ indicate a similar role in *C. neoformans*.

In summary, this work has provided the first family-wide insights into sirtuin function in the phylum Basidiomycota, and the first link between multiple sirtuins and virulence in a fungal pathogen. Perhaps most importantly, our complementation experiments highlighted a highly unusual and unanticipated phenotype in *C. neoformans* sirtuin mutants, the inability to restore wild-type phenotype even upon the reintroduction of the wild-type gene transcribed at wild-type levels. These observations point towards an exciting model of sirtuin function where temporal inactivation of enzymes of this family potentially enable the permanent alteration of the epigenetic state of the genome, and provide a powerful contribution to the microevolutionary burst without compromising the genome sequence. These findings have therefore set the stage for exciting further work that has the potential to integrate the acquisition of adaptive mutations alongside epigenetic change in the microevolutionary burst *C. neoformans* undergoes in the hostile host environment.

## Methods

### Bioinformatic analyses

Sirtuin gene identification was performed using BLASTP with validation using OrthMCL^62^ and sequence analyses performed using MacVector 10.0 (MacVector Inc., USA). with sequence alignments and protein similarity generated using MUSCLE. Phylogenetic trees were constructed using MEGA7^63^. A maximum likelihood tree was created with a WAG + G + I model and gamma shape parameters with 500 bootstrap replicates. Sequence traces generated at the Australian Genome Research Facility (Australia) were analyzed using Sequencher 4.7 (Gene Codes Corporation, USA).

### Strains and growth conditions

*C. neoformans* strains generated in this study are listed in Supplementary Table S3 and were cultured on YPD unless stated otherwise; fungal strains were stored in 15% glycerol at −80 °C until use. *E. coli* Mach1 cells (Invitrogen, USA) served as the host strain for transformation and propagation of all plasmids using lysogeny broth supplemented with 100 μg/mL ampicillin (Sigma, USA)^64^. Plasmids generated in this study are listed in Supplementary Table S4.

### Construction of *C. neoformans* mutant and complemented strains

Gene deletion constructs were generated using PCR overlap and biolistic transformation as previously described^65^. Briefly, the various constructs were prepared using overlap PCR, joining approximately 1 kb of the 5′ region of the gene of interest, the G418 resistance marker *NEO* and approximately 1 kb of the 3′ region; primers used are listed in Supplementary Table S5. H99 genomic DNA was used as the template for each sirtuin gene, and the plasmid pJAF1 for *NEO*^15^,^66^. Transformations were carried out *via* biolistic particle delivery onto media containing 100 μg/mL G418 (Sigma, USA). Stable transformants were confirmed to be correct *via* Southern blot. All mutant strains were created at least twice, from independent transformations.

To complement the sirtuin mutant strains, a fragment containing each gene with approximately 1 kb of the 5′ region and 1 kb of the 3′ region was amplified using Phusion High-Fidelity DNA polymerase (New England Biolabs, USA) and cloned into the nourseothricin resistance Safe Haven vector pSDMA25^44^ (Supplementary Table S4). For *SIR2*, a second complementation plasmid with ~2 kb of 5′ region was also created. Each plasmid was linearized using BaeI, AscI or PacI and biolistically transformed into the corresponding sirtuin mutant for complementation. Stable transformants were selected on YPD supplemented with 100 μg/mL nourseothricin and were confirmed to be correct *via* Southern blot.

To create a 3 × HA tagged version of *SIR2*, a 5-part overlap PCR was performed. Overlap primers were used to amplify and stitch together the final 1,016 bp of the *SIR2* coding region, the 3 × HA tag, 347 bp of the *SIR2* 3′ region, the *NEO* selectable marker, and lastly a final 771 bp fragment downstream from the previously utilized 3′ *SIR2* region (Supplementary Table S5). H99 genomic DNA was used as the template for the three *SIR2* components, the plasmid pJAF1 for *NEO*^15^,^66^ and a pre-ordered gBlock containing the 3 × HA sequence for the tag (IDT, USA). All PCRs were performed using Phusion High-Fidelity DNA polymerase (New England Biolabs, USA). The resulting 5-part construct was A-tailed with Taq polymerase then cloned into pCR2.1-TOPO (Invitrogen, USA) to create pSDMA24 and sequenced to confirm it was error free. The construct was cut out of pSDMA24 with KpnI and XmaI and biolistically transformed into *C. neoformans*, with stable transformants selected on 100 μg/mL G418. The resulting strain was confirmed to be correct *via* Southern blot.

### *In vitro* virulence factor assays

Strains were grown for 16 hr in YPD, washed in dH_2_O and diluted to OD_600_ 1.0, and 10-fold serially diluted prior to spotting. Melanization assays were performed on solid L-DOPA media supplemented with 10 mM asparagine^67^. Phospholipase B production was visualized on Sabouraud dextrose agar with 8% egg yolk^9^, protease production on complete Yeast Nitrogen Base (YNB) with 1% BSA^68^ and urease production on Christensen’s agar^69^. Capsule was induced by growth for 24 hr in RPMI 1640 media (Life Technologies, USA) with 2% glucose and 10% fetal bovine serum (Life Technologies, USA) and stained with India ink (BD Diagnostics, USA). Cells were imaged with a Leica DM2500 microscope and DFC425C camera (Leica, Germany. All assays were performed at both 30 and 37 °C.

### Phenotypic stress assays

Phenotypic plate assays were performed on YNB (without amino acids and ammonium sulfate) media supplemented with 2% glucose and 10 mM ammonium sulfate unless stated otherwise. For UV sensitivity, cells were spotted then exposed for 6 sec to UV light at 48 mJ/cm^2^ in a UV Stratalinker (Stratagene, USA). For all other assays, the stressor was added to the media immediately prior to pouring at the following concentrations: 0.001% SDS (Sigma, USA), 5 μg/mL fluconazole (Sigma, USA), 0.25 μg/mL itraconazole (Sigma, USA), 1 μg/mL fenpropimorph (Sigma, USA), 1 M NaCl (Sigma, USA), 1 M KCl (Sigma, USA), 125 mM *t*-butyl hydroperoxide (Sigma, USA), 1 mM NaNO_2_ (Sigma, USA), 1 μg/mL cyclohexamide (Sigma, USA) and 1 mM mercaptopurine (Sigma, USA). Tenfold serial dilutions were prepared immediately prior to testing, with plates being imaged at 24–96 hr. All assays were performed at both 30 and 37 °C.

### Matings

Mating assays were performed on Murashige-Skoog media (Sigma, USA) and V8 juice media plus 100 μg/mL *myo*-inositol (pH 5.0) (Sigma, USA) using KN99**a** as the *MAT**a*** partner. Strains were co-inoculated in equal quantities *via* toothpick and incubated in the dark at room temperature for two weeks. Filamentation and sporulation was examined and imaged using a Leica M125 stereomicroscope fitted with a Leica DFC 425c digital camera running Leica Application Suite 3.6 software (Leica, Germany).

### Mass spectrometry

To determine whole cell proteome changes, 50 mL cultures were grown to OD 0.8 in YNB (2% glucose, 10 mM ammonium sulfate) at 30 °C overnight. Cells were collected by centrifugation and washed twice with TBS. Collected cells were resuspended in protein lysis buffer (100 mM Tris pH 8.0, 0.1 mM EDTA) with the addition of protease inhibitors (1 mM phenylmethylsulfonyl fluoride (Sigma, USA), 1 × protease inhibitor cocktail (Roche, Switzerland)) and subjected to mechanical bead-beating with 0.5 mm glass beads for 1 min at 4 °C, followed by a 3 min rest, for a total of 8 cycles. Sample tubes were then suspended in 15 mL conical tubes, a hole punched in the base of the microcentrifuge tube, and centrifuged to pool lysate. Samples were denatured by addition of SDS (Sigma, USA) to a final concentration of 0.5% and DTT (Sigma, USA) to a final concentration of 10 mM, and incubated at 60 °C for 30 min. After cooling to room temperature, cysteines were alkylated by addition of acrylamide (Sigma, USA) to a final concentration of 50 mM and incubated at room temperature for 1 hr. Protein was precipitated by addition of 4 volumes 1:1 methanol:acetone and incubated at −20 °C for 16 hr, harvested *via* centrifugation, resuspended in Tris-HCl, pH 8 and digested with trypsin (Sigma, USA). Insoluble material was removed by centrifugation at 20,000× g for 10 min, and peptides were desalted with C18 ZipTips (Millipore, USA).

Peptides were analyzed as described previously^70^ by LC-ESI–MS/MS using a Prominence nanoLC system (Shimadzu, Japan) and TripleTOF 5600 mass spectrometry with a Nanospray III interface (Ab Sciex, USA). Identical LC conditions were used for SWATH-MS, with an MS-TOF scan from an *m*/*z* of 350–1,800 for 0.05 sec followed by high sensitivity information-independent acquisition with 26 *m*/*z* isolation windows with 1 *m*/*z* window overlap each for 0.1 sec across an *m*/*z* range of 400–1,250. Collision energy was automatically assigned by Analyst software (Ab Sciex, USA) based on *m*/*z* window ranges. Peptides were identified using ProteinPilot (Ab Sciex, USA), searching the UniProt database with standard settings. False discovery rate analysis using ProteinPilot was performed on all searches; peptides identified with greater than 99% confidence and with a local false discovery rate of less than 1% were included for further analysis. ProteinPilot search results were used as ion libraries for SWATH analyses. The abundance of proteins was measured automatically using PeakView (Ab Sciex, USA) with standard settings. Comparisons of protein relative abundance for the strains were performed with the MSstats package in R^71^.

Gene ontology enrichment was determined using a hypergeometric test within the GOstats package^72^ in R, using a *p*-value of <0.05 as cut-off for significance. A list of all proteins detected in proteomic analysis was used as the background for enrichment analysis. GO term annotation for *C. neoformans* was downloaded from UniProt.

### ChIP-Seq

100 mL of *C. neoformans* cells were grown to OD 0.8 in YNB (2% glucose, 10 mM ammonium sulfate) at 30 °C overnight with shaking and fixed for 20 min with a final concentration of 1% formaldehyde (Sigma USA), followed by quenching with a final concentration of 125 mM glycine (Sigma USA). Fixed cells were collected by centrifugation and washed with TBS + 125 mM glycine, followed by a second wash with TBS only. Cells were resuspended in buffer A (50 mM HEPES pH 7.5, 140 mM NaCl, 1 mM EDTA, 1% v/v Triton X-100, 0.1% w/v sodium deoxycholate) and protease inhibitors (1 mM PMSF and 1× protease cocktail inhibitor) and subjected to mechanical bead-beating with 0.5 mm zirconium silicate beads for 3 min at 4 °C, followed by a 1 min rest, for a total of 6 cycles. The resulting chromatin was then sheared by sonication in a Bioruptor Plus sonication device (Diagenode, USA) for 30 sec at full power output, followed by a 30 sec rest, for a total of 30 cycles. The lysate was clarified *via* centrifugation and protein concentration was determined with a DC protein assay (Bio-Rad, USA). 1 mg of protein was used for immunoprecipitation with 20 μL reserved as an input sample.

Dynabeads Protein B (Life Technologies, USA) were pre-hybridized in buffer B (50 mM HEPES pH 7.5, 500 mM NaCl, 1 mM EDTA, 1% (v/v) Triton X-100, 0.1% (w/v) sodium deoxycholate) with Mouse anti-HA antibody (Invitrogen, USA) for 1 hr at 4 °C with rotation on a Hula Mixer (Life Technologies, USA) prior to the addition of the protein sample, followed by incubation at 4 °C for an additional 2 hr. The protein-bound beads were sequentially washed with rotation for 5 min in 1 mL buffer A, buffer B (50 mM HEPES pH 7.5, 500 mM NaCl, 1 mM EDTA, 1% (v/v) Triton X-100, 0.1% (w/v) sodium deoxycholate), buffer C (10 mM Tris-HCl pH 8.0, 250 mM LiCl, 1 mM EDTA, 0.5% (v/v) NP-40, 0.5% w/v sodium deoxycholate), and buffer D (10 mM Tris, 1 mM EDTA), with immunoprecipitated protein eluted in buffer E (50 mM Tris pH 8.0, 10 mM EDTA, 1% (w/v) SDS). Chromatin from the input and IP samples was released by adding NaCl to a final concentration of 10 mM and incubating overnight at 65 °C. Samples were treated with RNase A for 30 min at 37 °C followed by pronase for 2 hr at 42 °C, and extracted using a QIAquick PCR Purification Kit (QIAGEN, Netherlands).

Purified ChIP-DNA from the input and IP samples was end repaired with Klenow DNA polymerase (New England Biolabs, USA) and purified using AMPure XP beads (Agencourt, USA). The samples were A-tailed using Klenow fragment (New England Biolabs, USA) before ligating Multiplex Oligos for Illumina (Index primer set 1) (Illumina, USA) using T4 DNA ligase (New England Biolabs, USA). Ligated indexed samples were subsequently amplified as per the kit instructions, and gel purified to remove adapter dimers and select optimal sizes (100 to 500 bp). Libraries were 7-way multiplexed on an Illumina MiSeq flow cell using MiSeq Reagent Kit v3 (Illumina, USA).

Reads generated from the input and IP samples were aligned to the *C. neoformans* type strain H99 genome reference sequence using Bowtie^73^. Peak calling was performed using MACS2, with a *p-*value of <0.01 considered statistically significant.

### *C. elegans* virulence assays

Starter cultures of strains were grown overnight at 30 °C in YPD (2% glucose) with shaking. 10 μL was subsequently spread on brain-heart infusion (BHI) and M9 minimal media plates^74^ and incubated overnight at 30 °C. Approximately 40 adult *C. elegans* worms were then transferred onto the *C. neoformans* inoculated plates and incubated at 25 °C, with worms examined visually using a Leica M125 stereomicroscope at 2.5 × magnification (Leica, Germany) at 24 hr intervals. Worms that did not respond to a touch with a platinum wire were considered dead. GraphPad Prism 7.0 was used to plot Kaplan-Meier survival curves, with the significance calculated using a log-rank test. *p-*value < 0.05 was considered significant.

### Murine survival assays

For murine infection assays, 6-week-old female BALB/c mice (Animal Resources Centre, Australia) were infected by nasal inhalation^75^. For each strain, 10 mice were inoculated with a 50 μL drop containing 5 × 10^5^ *C. neoformans* cells. A maximum of 5 mice were housed per individually ventilated cage (Tecniplast, USA) with Bed-o’Cobs 1/8″ bedding (The Andersons, USA), Crink-l’Nest nesting material (The Andersons, USA), and cardboard as environmental enrichment. Mice were provided Rat and Mouse Cubes (Specialty Feeds, Australia) and water *ad libitum*. Each mouse was examined and weighed twice daily for the duration of the experiment, with affected mice euthanized *via* CO_2_ inhalation once body weight had decreased to 80% of pre-infection weight or they exhibited symptoms consistent with infection. Death after CO_2_ inhalation was confirmed by pedal reflex. Kaplan-Meier survival curves were plotted using GraphPad Prism 7.0 (GraphPad Software, USA). Significance was analyzed using the log-rank test. *p-*values of <0.05 were considered significant.

### Ethics statement

This study was carried out in strict accordance with the recommendations in the Australian Code of Practice for the Care and Use of Animals for Scientific Purposes by the National Health and Medical Research Council (Australia). The protocol was approved by the Molecular Biosciences Animal Ethics Committee (AEC) of The University of Queensland (AEC approval no. SCMB/439/13/UQ/NHMRC). Infection was performed under methoxyflurane anesthesia, and all efforts were made to minimize suffering through adherence to the Guidelines to Promote the Wellbeing of Animals Used for Scientific Purposes as put forward by the National Health and Medical Research Council (Australia).

### Quantitative real-time PCR

*C. neoformans* strains were grown in YNB (2% glucose, 10 mM ammonium sulfate) with shaking at 30 °C for 16 hr. Cultures were harvested, cell pellets frozen and lyophilized, and total RNA isolated using TRIzol reagent (Life Technologies, USA). cDNA was generated using the Superscript III First-Strand Synthesis System (Invitrogen, USA). Quantitative real-time PCR (qRT-PCR) was performed using SYBR Green Supermix (Applied Biosystems, USA) and an Applied Biosystems 7900HT Fast Real Time PCR System with the following cycling conditions: denaturation at 95 °C for 10 min, followed by amplification and quantification in 45 cycles of 95 °C for 15 sec and 60 °C for 1 min, with melting-curve profiling at 95 °C for 2 min, 60 °C for 15 sec, and 95 °C for 15 sec. Relative gene expression was quantified using SDS 1.3.1 (Applied Biosystems, USA) based on the 2^−ΔΔCT^ method^76^. The actin-encoding gene *ACT1* (Supplementary Table S5) was used as a control for normalization. One-way analysis of variance was performed using the unpaired, two-tailed *t*-test in GraphPad Prism Version 7.0 (GraphPad Software, USA). *p-*values of <0.05 were considered statistically significant.

## Additional Information

**How to cite this article:** Arras, S. D. M. *et al*. Sirtuins in the phylum Basidiomycota: A role in virulence in *Cryptococcus neoformans. Sci. Rep.* **7**, 46567; doi: 10.1038/srep46567 (2017).

**Publisher's note:** Springer Nature remains neutral with regard to jurisdictional claims in published maps and institutional affiliations.

## Supplementary Material

Supplementary Information
