# Supplementary material for: Sirtuins in the phylum Basidiomycota: A role in virulence in Cryptococcus neoformans
Source: Sci Rep. 2017 Apr 21;7:46567. doi: 10.1038/srep46567 (PMC5399365; doi:10.1038/srep46567)
Supplement: Supplementary Information [file srep46567-s1.pdf]

Sirtuins in the phylum Basidiomycota: A role in virulence in *Cryptococcus neoformans*

Samantha D. M. Arras, Jessica L. Chitty, Maha S. I. Wizrah, Paige E. Erpf

Benjamin L. Schulz, Milos Tanurdzic and James A. Fraser

Supplementary Tables and Figures

Supplementary Table 1: % similarity

C. neoformans	*S. cerevisiae* Sir2	*S. cerevisiae* Hst1	*S. cerevisiae* Hst2	*S. cerevisiae* Hst3	*S. cerevisiae* Hst4	*C. albicans* Sir2 orf 19.1992	*C. albicans* Hst2 orf 19.2580	*C. albicans* Hst3 orf 19.1934	*C. albicans* orf 19.4761	*S. pombe* Sir2 SPBC16D10	*S. pombe* Hst2 SPCC132	*S. pombe* Hst4 SPAC1783	*A. nidulans* SirA AND 10449	*A. nidulans* AND 07461	*A. nidulans* AND 01226	*A. nidulans* AND 11872	*A. nidulans* AND 01782	*U. maydis* Hst1 UM00963	*U. maydis* Hst4 UM05239	*U. maydis* UM05758	*U. maydis* UM0589	*N. crassa* Nst1 NCU00523	*N. crassa* Nst2 NUC03059	*N. crassa* Nst3 NCU04737	*N. crassa* Nst4 NCU04859	*N. crassa* Nst5 NCU05973	*N. crassa* Nst7 NCU07624
Sir2	20.9 (53.2)	23.5 (59.9)	17.1 (44.6)	16.2 (43.8)	12.4 (35.2)	19.2 (49.1)	16.8 (42.9)	12.0 (34.3)	22.2 (57.0)	25.7 (64.3)	16.9 (44.4)	10.8 (31.6)	27.3 (68.2)	16.6 (43.5)	11.7 (32.6)	16.8 (43.3)	9.2 (27.0)	38.3 (91.4)	6.8 (24.1)	8.7 (24.7)	16.2 (42.8)	17.2 (45.8)	10.3 (30.8)	25.8 (66.6)	5.9 (16.3)	8.2 (27.2)	10.8 (31.7)
Hst2	17.1 (43.8)	19.3 (48.9)	35.9 (85.4)	18.6 (47.6)	14.6 (39.7)	16.9 (42.7)	36.7 (87.9)	13.9 (37.1)	15.5 (39.4)	18.9 (42.8)	34.0 (61.8)	12.6 (35.7)	18.1 (45.4)	33.2 (78.4)	11.2 (29.4)	28.6 (71.6)	15.2 (40.0)	14.0 (63.2)	10.9 (33.5)	6.9 (18.0)	39.9 (93.8)	32.4 (76.5)	12.3 (32.4)	15.0 (38.3)	5.7 (14.2)	13.2 (39.1)	14.2 (37.3)
Hst3	15.0 (37.2)	15.0 (38.6)	14.6 (40.8)	28.3 (70.9)	22.3 (58.0)	12.2 (31.5)	14.9 (38.1)	25.1 (64.0)	9.8 (25.8)	12.5 (32.7)	11.7 (38.6)	21.3 (51.5)	13.6 (35.0)	15.8 (40.0)	15.0 (40.9)	16.0 (40.7)	11.6 (32.7)	11.7 (31.7)	10.7 (31.8)	10.8 (26.7)	14.3 (37.3)	15.8 (38.8)	17.4 (45.4)	9.5 (25.9)	13.0 (30.5)	12.4 (33.7)	10.5 (28.6)
Hst4	11.1 (33.0)	11.5 (31.0)	14.2 (41.4)	25.0 (61.9)	20.1 (50.1)	9.4 (27.1)	13.7 (37.4)	15.4 (40.0)	8.5 (24.9)	10.9 (31.7)	13.0 (35.6)	20.6 (51.3)	11.0 (30.4)	12.8 (38.0)	16.2 (41.9)	13.1 (39.1)	10.0 (28.3)	11.3 (31.6)	7.5 (26.9)	11.4 (28.7)	11.8 (35.6)	12.7 (38.4)	17.4 (45.8)	10.3 (29.5)	8.0 (20.3)	11.9 (33.7)	13.6 (38.1)
Hst5	10.2 (27.6)	11.6 (32.2)	14.2 (40.0)	13.4 (36.7)	12.7 (38.6)	8.4 (26.4)	12.0 (34.8)	8.8 (26.6)	7.3 (22.7)	9.4 (28.1)	6.7 (36.6)	10.4 (30.1)	9.6 (29.0)	12.1 (38.2)	7.8 (23.0)	13.2 (39.8)	12.7 (36.9)	6.7 (22.8)	36.5 (89.4)	4.4 (14.3)	9.9 (31.7)	14.4 (40.7)	7.7 (23.2)	7.5 (22.5)	3.7 (10.4)	14.0 (40.5)	10.9 (30.5)

Supplementary Table 2

*sir2*Δ protein abundance increase

Gene to GO BP test for over-representation						
**GOBPID**	**Pvalue**	**OddsRatio**	**ExpCount**	**Count**	**Size**	**Term**
**GO:0006730**	0.002	Inf	0	3	3	one-carbon metabolic process

*sir2*Δ protein abundance decrease

Gene to GO BP test for over-representation						
**GOBPID**	**Pvalue**	**OddsRatio**	**ExpCount**	**Count**	**Size**	**Term**
**GO:0005975**	0.005	8.736	1	4	33	carbohydrate metabolic process
**GO:0044723**	0.010	9.310	1	3	21	single-organism carbohydrate metabolic process
**GO:0044262**	0.017	14.357	0	2	9	cellular carbohydrate metabolic process
**GO:0006430**	0.024	Inf	0	1	1	lysyl-tRNA aminoacylation
**GO:0046487**	0.024	Inf	0	1	1	glyoxylate metabolic process
**GO:0006097**	0.024	Inf	0	1	1	glyoxylate cycle
**GO:0046165**	0.024	Inf	0	1	1	alcohol biosynthetic process
**GO:0006020**	0.024	Inf	0	1	1	inositol metabolic process
**GO:0006021**	0.024	Inf	0	1	1	inositol biosynthetic process
**GO:0046173**	0.024	Inf	0	1	1	polyol biosynthetic process
**GO:0019751**	0.047	45.333	0	1	2	polyol metabolic process
**GO:0008654**	0.047	45.333	0	1	2	phospholipid biosynthetic process
**GO:0034637**	0.047	45.333	0	1	2	cellular carbohydrate biosynthetic process
**GO:0006012**	0.047	45.333	0	1	2	galactose metabolic process

*hst2*Δ protein abundance increase

**GOBPID**	**Pvalue**	**OddsRatio**	**ExpCount**	**Count**	**Size**	**Term**
**GO:0006446**	0.012	18.273	0	2	6	regulation of translational initiation
**GO:0006413**	0.017	14.582	0	2	7	translational initiation
**GO:0006417**	0.017	14.582	0	2	7	regulation of translation
**GO:0034248**	0.017	14.582	0	2	7	regulation of cellular amide metabolic process
**GO:0010608**	0.017	14.582	0	2	7	posttranscriptional regulation of gene expression
**GO:0001731**	0.017	14.582	0	2	7	formation of translation preinitiation complex
**GO:0032268**	0.017	14.582	0	2	7	regulation of cellular protein metabolic process
**GO:0022618**	0.028	10.364	0	2	9	ribonucleoprotein complex assembly
**GO:0051246**	0.028	10.364	0	2	9	regulation of protein metabolic process
**GO:0044262**	0.028	10.364	0	2	9	cellular carbohydrate metabolic process
**GO:0071826**	0.028	10.364	0	2	9	ribonucleoprotein complex subunit organization
**GO:0010468**	0.028	10.364	0	2	9	regulation of gene expression
**GO:0006694**	0.031	Inf	0	1	1	steroid biosynthetic process
**GO:0006422**	0.031	Inf	0	1	1	aspartyl-tRNA aminoacylation

**GO:0070084**	0.031	Inf	0	1	1	protein initiator methionine removal
**GO:0006430**	0.031	Inf	0	1	1	lysyl-tRNA aminoacylation
**GO:0008202**	0.031	Inf	0	1	1	steroid metabolic process
**GO:0046487**	0.031	Inf	0	1	1	glyoxylate metabolic process
**GO:0006097**	0.031	Inf	0	1	1	glyoxylate cycle
**GO:0009889**	0.035	9.045	0	2	10	regulation of biosynthetic process
**GO:0031326**	0.035	9.045	0	2	10	regulation of cellular biosynthetic process
**GO:0010556**	0.035	9.045	0	2	10	regulation of macromolecule biosynthetic process
**GO:2000112**	0.035	9.045	0	2	10	regulation of cellular macromolecule biosynthetic process
**GO:0051171**	0.042	8.020	0	2	11	regulation of nitrogen compound metabolic process
**GO:0031323**	0.042	8.020	0	2	11	regulation of cellular metabolic process
**GO:0060255**	0.049	7.200	0	2	12	regulation of macromolecule metabolic process

*hst2*Δ protein abundance decrease

**Gene to GO BP test for over-representation**						
**GOBPID**	**Pvalue**	**OddsRatio**	**ExpCount**	**Count**	**Size**	**Term**
**GO:0046040**	0.004	27.366	0	3	4	IMP metabolic process
**GO:0006188**	0.004	27.366	0	3	4	IMP biosynthetic process
**GO:0006189**	0.004	27.366	0	3	4	'de novo' IMP biosynthetic process
**GO:0034641**	0.007	2.372	22	30	208	cellular nitrogen compound metabolic process
**GO:1901564**	0.008	2.372	23	31	219	organonitrogen compound metabolic process
**GO:0044271**	0.012	2.158	17	24	158	cellular nitrogen compound biosynthetic process
**GO:0043603**	0.027	2.012	12	18	114	cellular amide metabolic process
**GO:0044724**	0.028	4.588	1	4	12	single-organism carbohydrate catabolic process
**GO:0006090**	0.028	4.588	1	4	12	pyruvate metabolic process
**GO:0044281**	0.030	1.925	17	23	159	small molecule metabolic process
**GO:0006730**	0.030	17.810	0	2	3	one-carbon metabolic process
**GO:0043043**	0.030	1.994	11	17	107	peptide biosynthetic process
**GO:0006412**	0.030	1.994	11	17	107	translation
**GO:0006518**	0.033	1.965	11	17	108	peptide metabolic process
**GO:0016052**	0.037	4.067	1	4	13	carbohydrate catabolic process
**GO:0043604**	0.040	1.909	12	17	110	amide biosynthetic process
**GO:0034645**	0.041	1.878	12	18	119	cellular macromolecule biosynthetic process
**GO:0009167**	0.045	2.540	3	7	33	purine ribonucleoside monophosphate metabolic process
**GO:0009126**	0.045	2.540	3	7	33	purine nucleoside monophosphate metabolic process
**GO:0009059**	0.045	1.853	13	18	120	macromolecule biosynthetic process
**GO:0006807**	0.045	1.900	26	32	251	nitrogen compound metabolic

						process
**GO:1901135**	0.046	2.157	6	10	55	carbohydrate derivative metabolic process
**GO:0072524**	0.047	3.077	2	5	20	pyridine-containing compound metabolic process

*hst3*Δ protein abundance increase

Gene to GO BP test for over-representation						
**GOBPID**	**Pvalue**	**OddsRatio**	**ExpCount**	**Count**	**Size**	**Term**
**GO:0044281**	0.000	2.953	27	43	159	small molecule metabolic process
**GO:0006082**	0.000	2.985	17	30	97	organic acid metabolic process
**GO:0044710**	0.000	2.712	31	46	183	single-organism metabolic process
**GO:0019752**	0.000	2.818	16	29	96	carboxylic acid metabolic process
**GO:0043436**	0.000	2.818	16	29	96	oxoacid metabolic process
**GO:0044712**	0.000	6.057	3	10	19	single-organism catabolic process
**GO:1901575**	0.000	3.680	7	16	41	organic substance catabolic process
**GO:0009056**	0.000	3.680	7	16	41	catabolic process
**GO:0044763**	0.000	2.536	35	48	201	single-organism cellular process
**GO:0044699**	0.000	2.536	37	50	214	single-organism process
**GO:0044724**	0.001	7.366	2	7	12	single-organism carbohydrate catabolic process
**GO:0044248**	0.002	4.144	4	10	23	cellular catabolic process
**GO:0016052**	0.002	6.121	2	7	13	carbohydrate catabolic process
**GO:0006006**	0.004	20.353	1	4	5	glucose metabolic process
**GO:0005975**	0.005	3.105	6	12	33	carbohydrate metabolic process
**GO:0006091**	0.005	3.296	5	11	29	generation of precursor metabolites and energy
**GO:1901605**	0.010	2.692	6	12	36	alpha-amino acid metabolic process
**GO:0044283**	0.015	2.273	9	15	51	small molecule biosynthetic process
**GO:0046394**	0.015	2.416	7	13	42	carboxylic acid biosynthetic process
**GO:0016053**	0.015	2.416	7	13	42	organic acid biosynthetic process
**GO:1901607**	0.015	2.784	5	10	29	alpha-amino acid biosynthetic process
**GO:0006165**	0.016	5.104	2	5	10	nucleoside diphosphate phosphorylation
**GO:0006757**	0.016	5.104	2	5	10	ATP generation from ADP
**GO:0006096**	0.016	5.104	2	5	10	glycolytic process
**GO:0046939**	0.016	5.104	2	5	10	nucleotide phosphorylation
**GO:0009082**	0.019	6.745	1	4	7	branched-chain amino acid biosynthetic process
**GO:0044282**	0.019	6.745	1	4	7	small molecule catabolic process
**GO:0044265**	0.020	3.852	2	6	14	cellular macromolecule catabolic process
**GO:0044257**	0.020	3.852	2	6	14	cellular protein catabolic process
**GO:0051603**	0.020	3.852	2	6	14	proteolysis involved in cellular protein catabolic process
**GO:0019362**	0.022	3.290	3	7	18	pyridine nucleotide metabolic process
**GO:0046496**	0.022	3.290	3	7	18	nicotinamide nucleotide metabolic process

**GO:0006733**	0.022	3.290	3	7	18	oxidoreduction coenzyme metabolic process
**GO:0006520**	0.023	2.023	11	17	63	cellular amino acid metabolic process
**GO:0046031**	0.026	4.241	2	5	11	ADP metabolic process
**GO:0009185**	0.026	4.241	2	5	11	ribonucleoside diphosphate metabolic process
**GO:0009179**	0.026	4.241	2	5	11	purine ribonucleoside diphosphate metabolic process
**GO:0009132**	0.026	4.241	2	5	11	nucleoside diphosphate metabolic process
**GO:0009135**	0.026	4.241	2	5	11	purine nucleoside diphosphate metabolic process
**GO:0008652**	0.029	2.323	6	11	36	cellular amino acid biosynthetic process
**GO:0006508**	0.029	3.414	3	6	15	proteolysis
**GO:0006549**	0.029	Inf	0	2	2	isoleucine metabolic process
**GO:0009097**	0.029	Inf	0	2	2	isoleucine biosynthetic process
**GO:0019725**	0.033	5.044	1	4	8	cellular homeostasis
**GO:0042592**	0.033	5.044	1	4	8	homeostatic process
**GO:0009081**	0.033	5.044	1	4	8	branched-chain amino acid metabolic process
**GO:0000096**	0.033	5.044	1	4	8	sulfur amino acid metabolic process
**GO:0000097**	0.033	5.044	1	4	8	sulfur amino acid biosynthetic process
**GO:0005996**	0.033	5.044	1	4	8	monosaccharide metabolic process
**GO:0019318**	0.033	5.044	1	4	8	hexose metabolic process
**GO:0045454**	0.033	5.044	1	4	8	cell redox homeostasis
**GO:0009205**	0.034	2.466	5	9	28	purine ribonucleoside triphosphate metabolic process
**GO:0009199**	0.034	2.466	5	9	28	ribonucleoside triphosphate metabolic process
**GO:0009144**	0.034	2.466	5	9	28	purine nucleoside triphosphate metabolic process
**GO:0006163**	0.035	2.226	6	11	37	purine nucleotide metabolic process
**GO:0072521**	0.038	2.113	7	12	42	purine-containing compound metabolic process
**GO:0006090**	0.039	3.625	2	5	12	pyruvate metabolic process
**GO:0009057**	0.039	2.767	3	7	20	macromolecule catabolic process
**GO:0072524**	0.039	2.767	3	7	20	pyridine-containing compound metabolic process

*hst3*Δ protein abundance decrease

Gene to GO BP test for over-representation						
**GOBPID**	**Pvalue**	**OddsRatio**	**ExpCount**	**Count**	**Size**	**Term**
**GO:0051649**	0.008	5.719	1	5	13	establishment of localization in cell
**GO:0046907**	0.008	5.719	1	5	13	intracellular transport
**GO:1902582**	0.010	13.286	1	3	5	single-organism intracellular transport
**GO:1901360**	0.010	2.253	13	20	118	organic cyclic compound metabolic process
**GO:0043038**	0.011	5.069	2	5	14	amino acid activation
**GO:0043039**	0.011	5.069	2	5	14	tRNA aminoacylation
**GO:0006418**	0.011	5.069	2	5	14	tRNA aminoacylation for protein

						translation
**GO:0051641**	0.011	5.069	2	5	14	cellular localization
**GO:0046700**	0.011	Inf	0	2	2	heterocycle catabolic process
**GO:1901361**	0.011	Inf	0	2	2	organic cyclic compound catabolic process
**GO:0046483**	0.016	2.146	12	19	114	heterocycle metabolic process
**GO:0016482**	0.018	8.833	1	3	6	cytoplasmic transport
**GO:0006399**	0.020	4.125	2	5	16	tRNA metabolic process
**GO:0006725**	0.028	1.986	12	18	112	cellular aromatic compound metabolic process
**GO:0044270**	0.032	17.349	0	2	3	cellular nitrogen compound catabolic process
**GO:0034660**	0.034	3.471	2	5	18	ncRNA metabolic process
**GO:0006139**	0.036	1.965	11	16	98	nucleobase-containing compound metabolic process

*hst4*Δ protein abundance increase

Gene to GO BP test for over-representation						
GOBPID	Pvalue	OddsRatio	ExpCount	Count	Size	Term
**GO:0006725**	0.005	2.065	21	31	112	cellular aromatic compound metabolic process
**GO:0046483**	0.007	2.000	21	31	114	heterocycle metabolic process
**GO:0006139**	0.011	1.967	18	27	98	nucleobase-containing compound metabolic process
**GO:1901360**	0.012	1.878	22	31	118	organic cyclic compound metabolic process
**GO:0034641**	0.019	1.742	39	48	208	cellular nitrogen compound metabolic process
**GO:0042558**	0.035	Inf	0	2	2	pteridine-containing compound metabolic process
**GO:0006760**	0.035	Inf	0	2	2	folic acid-containing compound metabolic process
**GO:0009225**	0.035	Inf	0	2	2	nucleotide-sugar metabolic process
**GO:0055086**	0.036	1.894	11	17	60	nucleobase-containing small molecule metabolic process
**GO:0006950**	0.038	3.761	2	5	11	response to stress
**GO:0006753**	0.042	1.919	10	15	52	nucleoside phosphate metabolic process
**GO:0009117**	0.042	1.919	10	15	52	nucleotide metabolic process

*hst4*Δ protein abundance decrease

Gene to GO BP test for over-representation						
GOBPID	Pvalue	OddsRatio	ExpCount	Count	Size	Term
**GO:0006555**	0.002	14.362	1	4	7	methionine metabolic process
**GO:0009086**	0.002	14.362	1	4	7	methionine biosynthetic process
**GO:0072521**	0.003	3.750	4	10	42	purine-containing compound metabolic process
**GO:0006790**	0.003	6.099	2	6	17	sulfur compound metabolic process
**GO:0042278**	0.003	3.922	3	9	36	purine nucleoside metabolic process
**GO:0046128**	0.003	3.922	3	9	36	purine ribonucleoside metabolic process
**GO:0000096**	0.003	10.743	1	4	8	sulfur amino acid metabolic process

**GO:0000097**	0.003	10.743	1	4	8	sulfur amino acid biosynthetic process
**GO:0044763**	0.004	2.664	19	27	201	single-organism cellular process
**GO:0044281**	0.004	2.579	15	23	159	small molecule metabolic process
**GO:0009119**	0.005	3.631	4	9	38	ribonucleoside metabolic process
**GO:0044710**	0.006	2.509	17	25	183	single-organism metabolic process
**GO:1901657**	0.007	3.377	4	9	40	glycosyl compound metabolic process
**GO:0009116**	0.007	3.377	4	9	40	nucleoside metabolic process
**GO:0044272**	0.008	5.441	1	5	15	sulfur compound biosynthetic process
**GO:0006793**	0.009	2.742	6	12	65	phosphorus metabolic process
**GO:0009205**	0.010	3.740	3	7	28	purine ribonucleoside triphosphate metabolic process
**GO:0009199**	0.010	3.740	3	7	28	ribonucleoside triphosphate metabolic process
**GO:0009144**	0.010	3.740	3	7	28	purine nucleoside triphosphate metabolic process
**GO:1901605**	0.012	3.244	3	8	36	alpha-amino acid metabolic process
**GO:0044699**	0.013	2.322	20	27	214	single-organism process
**GO:0044248**	0.014	3.882	2	6	23	cellular catabolic process
**GO:0006163**	0.014	3.123	3	8	37	purine nucleotide metabolic process
**GO:0009141**	0.014	3.395	3	7	30	nucleoside triphosphate metabolic process
**GO:0006796**	0.017	2.534	6	11	62	phosphate-containing compound metabolic process
**GO:0009066**	0.018	5.314	1	4	12	aspartate family amino acid metabolic process
**GO:0009067**	0.018	5.314	1	4	12	aspartate family amino acid biosynthetic process
**GO:0044282**	0.020	7.833	1	3	7	small molecule catabolic process
**GO:0044237**	0.023	3.354	31	36	333	cellular metabolic process
**GO:0044712**	0.024	3.845	2	5	19	single-organism catabolic process
**GO:0019509**	0.024	20.486	0	2	3	L-methionine biosynthetic process from methylthioadenosine
**GO:0071265**	0.024	20.486	0	2	3	L-methionine biosynthetic process
**GO:0071267**	0.024	20.486	0	2	3	L-methionine salvage
**GO:0043102**	0.024	20.486	0	2	3	amino acid salvage
**GO:0009201**	0.025	4.711	1	4	13	ribonucleoside triphosphate biosynthetic process
**GO:0009206**	0.025	4.711	1	4	13	purine ribonucleoside triphosphate biosynthetic process
**GO:0009142**	0.025	4.711	1	4	13	nucleoside triphosphate biosynthetic process
**GO:0009145**	0.025	4.711	1	4	13	purine nucleoside triphosphate biosynthetic process
**GO:0046034**	0.025	3.273	2	6	26	ATP metabolic process
**GO:0072522**	0.025	3.273	2	6	26	purine-containing compound biosynthetic process
**GO:1901575**	0.026	2.714	4	8	41	organic substance catabolic process
**GO:0009056**	0.026	2.714	4	8	41	catabolic process
**GO:0009150**	0.033	2.750	3	7	35	purine ribonucleotide metabolic

						process
**GO:0009987**	0.034	5.758	34	38	368	cellular process
**GO:0042451**	0.036	3.346	2	5	21	purine nucleoside biosynthetic process
**GO:0046129**	0.036	3.346	2	5	21	purine ribonucleoside biosynthetic process
**GO:0019637**	0.036	2.276	6	10	60	organophosphate metabolic process
**GO:0055086**	0.036	2.276	6	10	60	nucleobase-containing small molecule metabolic process
**GO:0016310**	0.041	3.834	1	4	15	phosphorylation
**GO:0006082**	0.041	2.004	9	14	97	organic acid metabolic process
**GO:0090407**	0.042	2.822	3	6	29	organophosphate biosynthetic process
**GO:1901607**	0.042	2.822	3	6	29	alpha-amino acid biosynthetic process
**GO:0006164**	0.043	3.140	2	5	22	purine nucleotide biosynthetic process
**GO:0009259**	0.044	2.552	3	7	37	ribonucleotide metabolic process
**GO:0006644**	0.045	10.216	0	2	4	phospholipid metabolic process
**GO:0006637**	0.045	10.216	0	2	4	acyl-CoA metabolic process
**GO:0015991**	0.045	10.216	0	2	4	ATP hydrolysis coupled proton transport
**GO:0015988**	0.045	10.216	0	2	4	energy coupled proton transmembrane transport, against electrochemical gradient
**GO:0035383**	0.045	10.216	0	2	4	thioester metabolic process
**GO:0006084**	0.045	10.216	0	2	4	acetyl-CoA metabolic process
**GO:0006520**	0.050	2.128	6	10	63	cellular amino acid metabolic process

*hst5*Δ protein abundance increase

Gene to GO BP test for over-representation						
**GOBPID**	**Pvalue**	**OddsRatio**	**ExpCount**	**Count**	**Size**	**Term**
**GO:0043603**	0.002	2.682	12	21	113	cellular amide metabolic process
**GO:0043043**	0.006	2.411	11	19	106	peptide biosynthetic process
**GO:0006412**	0.006	2.411	11	19	106	translation
**GO:0006518**	0.007	2.375	11	19	107	peptide metabolic process
**GO:0043604**	0.009	2.306	12	19	109	amide biosynthetic process
**GO:0034645**	0.010	2.253	13	20	118	cellular macromolecule biosynthetic process
**GO:0009059**	0.011	2.222	13	20	119	macromolecule biosynthetic process
**GO:0006544**	0.011	Inf	0	2	2	glycine metabolic process
**GO:1901564**	0.013	2.191	24	31	219	organonitrogen compound metabolic process
**GO:0010467**	0.018	2.077	13	20	124	gene expression
**GO:0006730**	0.032	17.349	0	2	3	one-carbon metabolic process
**GO:0006807**	0.035	1.968	27	33	251	nitrogen compound metabolic process

*hst5*Δ protein abundance decrease

Gene to GO BP test for over-representation						
**GOBPID**	**Pvalue**	**OddsRatio**	**ExpCount**	**Count**	**Size**	**Term**
**GO:0044763**	0.003	5.365	8	14	201	single-organism cellular process
**GO:0044699**	0.007	4.713	9	14	214	single-organism process

**GO:0009112**	0.014	17.733	0	2	5	nucleobase metabolic process
**GO:0046112**	0.014	17.733	0	2	5	nucleobase biosynthetic process
**GO:0044710**	0.021	3.242	7	12	183	single-organism metabolic process
**GO:0016570**	0.041	Inf	0	1	1	histone modification
**GO:0045936**	0.041	Inf	0	1	1	negative regulation of phosphate metabolic process
**GO:0044042**	0.041	Inf	0	1	1	glucan metabolic process
**GO:0051174**	0.041	Inf	0	1	1	regulation of phosphorus metabolic process
**GO:0000272**	0.041	Inf	0	1	1	polysaccharide catabolic process
**GO:0051261**	0.041	Inf	0	1	1	protein depolymerization
**GO:0010563**	0.041	Inf	0	1	1	negative regulation of phosphorus metabolic process
**GO:0006112**	0.041	Inf	0	1	1	energy reserve metabolic process
**GO:0005980**	0.041	Inf	0	1	1	glycogen catabolic process
**GO:0006140**	0.041	Inf	0	1	1	regulation of nucleotide metabolic process
**GO:0005977**	0.041	Inf	0	1	1	glycogen metabolic process
**GO:0018208**	0.041	Inf	0	1	1	peptidyl-proline modification
**GO:0030042**	0.041	Inf	0	1	1	actin filament depolymerization
**GO:0044275**	0.041	Inf	0	1	1	cellular carbohydrate catabolic process
**GO:1902224**	0.041	Inf	0	1	1	ketone body metabolic process
**GO:0044247**	0.041	Inf	0	1	1	cellular polysaccharide catabolic process
**GO:0046487**	0.041	Inf	0	1	1	glyoxylate metabolic process
**GO:0006097**	0.041	Inf	0	1	1	glyoxylate cycle
**GO:0019220**	0.041	Inf	0	1	1	regulation of phosphate metabolic process
**GO:0000412**	0.041	Inf	0	1	1	histone peptidyl-prolyl isomerization
**GO:0000413**	0.041	Inf	0	1	1	protein peptidyl-prolyl isomerization
**GO:0046950**	0.041	Inf	0	1	1	cellular ketone body metabolic process
**GO:0046952**	0.041	Inf	0	1	1	ketone body catabolic process
**GO:0009251**	0.041	Inf	0	1	1	glucan catabolic process
**GO:0006073**	0.041	Inf	0	1	1	cellular glucan metabolic process
**GO:0045980**	0.041	Inf	0	1	1	negative regulation of nucleotide metabolic process
**GO:0016568**	0.041	Inf	0	1	1	chromatin modification
**GO:0016569**	0.041	Inf	0	1	1	covalent chromatin modification
**GO:0009156**	0.047	4.571	1	3	21	ribonucleoside monophosphate biosynthetic process
**GO:0009124**	0.047	4.571	1	3	21	nucleoside monophosphate biosynthetic process
**GO:1902589**	0.047	7.524	0	2	9	single-organism organelle organization
**GO:0044262**	0.047	7.524	0	2	9	cellular carbohydrate metabolic process

Supplementary Table 3: Fungal strains used in this study

Strain	Genotype	Original source
H99	Wild-type	Genome ref
$H99_O$	Wild-type	Genome ref
sir2Δ	*SIR2* deletion (mutant 1) in H99 background	This study
sir2Δ+SIR2	*sir2Δ* complemented with *SIR2* in Safe Haven (1 kb promoter)	This study
sir2Δ	*SIR2* deletion (mutant 2) in H99 background	This study
sir2Δ+SIR2	*sir2Δ* complemented with *SIR2* in Safe Haven (1 kb promoter)	This study
sir2Δ	*SIR2* deletion (mutant 3) in $H99_O$ background	This study
sir2Δ+SIR2	*sir2Δ* complemented with *SIR2* in Safe Haven (1 kb promoter)	This study
sir2Δ+SIR2	*sir2Δ* complemented with *SIR2* in Safe Haven (2 kb promoter)	This study
sir2Δ	*SIR2* deletion (mutant 4) in $H99_O$ background	This study
sir2Δ+SIR2	*sir2Δ* complemented with *SIR2* in Safe Haven (1 kb promoter)	This study
sir2Δ+SIR2	*sir2Δ* complemented with *SIR2* in Safe Haven (2 kb promoter)	This study
hst2Δ	*HST2* deletion in H99 background	This study
hst2Δ+HST2	*hst2Δ* complemented with *HST2* in Safe Haven	This study
hst3Δ	*HST3* deletion in H99 background	This study
hst3Δ+HST3	*hst3Δ* complemented with *HST3* in Safe Haven	This study
hst4Δ	*HST4* deletion in H99 background	This study
hst4Δ+HST4	*hst4Δ* complemented with *HST4* in Safe Haven	This study
hst5Δ	*HST5* deletion in H99 background	This study
hst5Δ+HST5	*hst5Δ* complemented with *HST5* in Safe Haven	This study
SIR2:HA	*SIR2* HA tagged in native location	This study

Supplementary Table 4: Plasmids used in this study

Strain	Description	Original source
pJAF1	*NEO* resistance cassette vector	Fraser ref
pSDMA25	*NAT* vector for targeted integration at Safe Haven	Arras ref
pSDMA48	*SIR2* (1 kb promoter) in pSDMA25	This study
pSDMA69	*SIR2* (2 kb promoter) in pSDMA25	This study
pSDMA50	*HST2* in pSDMA25	This study
pSDMA49	*HST3* in pSDMA25	This study
pSDMA52	*HST4* in pSDMA25	This study
pSDMA53	*HST5* in pSDMA25	This study
pBluescript –SK	phagemid vector	This study
pSDMA24	*SIR2:HA* in pBluescript –SK	This study

Supplementary Table 5: Primers used in this study

	Primer name	Sequence
**UQ2198**	*SIR2* sequencing primer #1	GCCTTCCTCTGCCTCGCCTAC
**UQ2199**	*SIR2* sequencing primer #2	GGTTCACCTCCTCCCCATTCA
**UQ2200**	*SIR2* sequencing primer #3	CGAGATTCCTGCCCCAGAGAG
**UQ2201**	*SIR2* sequencing primer #4	GCCAATTAAAGCCAAGACCGT
**UQ2203**	*SIR2* sequencing primer #6	TACGGAGGATATGGCGGGAGA
**UQ2204**	*HST5* sequencing primer #1	CGTCGATAGCGGAATTAGAGC
**UQ2205**	*HST5* sequencing primer #2	CACAACCCAACCCAACACATA
**UQ2206**	*HST5* sequencing primer #3	CGCAACGCTACAGGGTGAAAC
**UQ2207**	*HST5* sequencing primer #4	AAAACCCACAGAATCGCAACT
**UQ2209**	*HST5* sequencing primer #5	CCCCCAGCAAGAACATCCATC
**UQ2210**	*HST4* sequencing primer #1	TGCTCATGGTTCAACGGGATT
**UQ2211**	*HST4* sequencing primer #2	ATCCGACACCCAAAAATCATC
**UQ2212**	*HST4* sequencing primer #3	CACCCAACGCTTCTCGCAAAA
**UQ2213**	*HST4* sequencing primer #4	CGGTCACGAAAAAGCGGTACA
**UQ2214**	*HST4* sequencing primer #5	CACACCGACGAGGAAGGATGA
**UQ2217**	*HST2* sequencing primer #1	GAACTGGCGATGCGTGACAAC
**UQ2218**	*HST2* sequencing primer #2	TCCCAGAAGCGGTATTTGAGC
**UQ2219**	*HST2* sequencing primer #3	GTTCAACCCTTCGCCTCTCTC
**UQ2220**	*HST2* sequencing primer #4	TCAAATTATCACGCGGCAACT
**UQ2221**	*HST2* sequencing primer #5	TTCCTGCCCTTCATCTTCTTT
**UQ2229**	*HST5* 5' upper	ACTGGATGGGTTTTGGGAAGT
**UQ2230**	*HST5* 5' lower	AGCTCACATCCTCGCAGCACTGAGGCATATCCTAGT
**UQ2231**	*HST5* 3' upper	TGTTAATACAGATAAACCTGAAGATTTAGACAGTAA
**UQ2232**	*HST5* 3' lower	ATTAAGCCTGCCAACATTCTC
**UQ2234**	*HST2* 5' upper	GCTTTCAAGTGCAGGTTGTTT
**UQ2235**	*HST2* 5' lower	AGCTCACATCCTCGCAGCCAGAATCAGAAGTCCCGT
**UQ2236**	*HST2* 3' upper	TGTTAATACAGATAAACCAGGAAAGAAAGAAGCAG
**UQ2237**	*HST2* 3' lower	GGCTGAATTGGATGGTAAAGT
**UQ2239**	*HST3* 5' upper	GGAGCAGGCTAGGTGAGGAGT
**UQ2240**	*HST3* 5' lower	AGCTCACATCCTCGCAGCGCCCGCGTTGTTGATGTC
**UQ2241**	*HST3* 3' upper	TGTTAATACAGATAAACCAGGACAATGCAGATGTAT
**UQ2244**	*HST4* 5' upper	GAAGGCGATGATTGAGATAGA
**UQ2245**	*HST4* 5' lower	AGCTCACATCCTCGCAGCGTTAGGATACTCTTCGAC
**UQ2246**	*HST4* 3' upper	TGTTAATACAGATAAACCTCTGTTAGATCGTACTCG
**UQ2247**	*HST4* 3' lower	AAAAGCGTTCTTGCGTTCCTA
**UQ2248**	*SIR2* 5' upper	AGCCAGCGGTATTTTGAAAGT
**UQ2249**	*SIR2* 5' lower	AGCTCACATCCTCGCAGCTAAGCTAACGAGTTCAAG
**UQ2250**	*SIR2* 3' upper	TGTTAATACAGATAAACCGGACTTGTTTCAAGCCCA

**UQ2251**	*SIR2* 3’ lower	ATCTCCTACCCTCCCTCTGTG
**UQ2345**	*HST3* KO 3’ lower	GTATATAGCGGGCCACACTAC
**UQ2362**	*SIR2* NEO upper	CGTTAGCTTAGCTGCGAGGATGTGAGCTGGAGAGCG
**UQ2363**	*SIR2* NEO lower	TGGGCTTGAAACAAGTCCGGTTTATCTGTATTAACA
**UQ2364**	*HST2* NEO upper	ACGGGACTTCTGATTCTGGCTGCGAGGATGTGAGCT
**UQ2365**	*HST2* NEO lower	CCTGCTTCTTTCTTTCCTGGTTTATCTGTATTAACA
**UQ2366**	*HST3* NEO upper	GACATCAACAACGCGGGCGCTGCGAGGATGTGAGCT
**UQ2367**	*HST3* NEO lower	ATACATCTGCATTGTCCTGGTTTATCTGTATTAACA
**UQ2368**	*HST4* NEO upper	GTCGAAGAGTATCCTAACGCTGCGAGGATGTGAGCT
**UQ2369**	*HST4* NEO lower	CGAGTACGATCTAACAGAGGTTTATCTGTATTAACA
**UQ2370**	*HST5* NEO upper	ACTAGGATATGCCTCAGTGCTGCGAGGATGTGAGCT
**UQ2371**	*HST5* NEO lower	TTACTGTCTAAATCTTCAGGTTTATCTGTATTAACA
**UQ2697**	*SIR2:HA* 5’ upper	GAACCCGGTATCATCAAGGTG
**UQ2698**	*SIR2:HA* 5’ lower	AACATCGTATGGGTAACCTGAACTTGCATTCCCAAGCA
**UQ2699**	*SIR2:HA* HA tag upper	TGCTTGGGAATGCAAGTTCAGGTTACCCATACGATGTT
**UQ2700**	*SIR2:HA* HA tag lower	AATGGGCTTGAAACAAGTCCTCAAGCAGCGTAATCTGG
**UQ2701**	*SIR2:HA* terminator upper	CCAGATTACGCTGCTTGAGGACTTGTTTCAAGCCCATT
**UQ2702**	*SIR2:HA* terminator lower	CAGCTCACATCCTCGCAGCGTAGTATCATGGCTTCAGG
**UQ2703**	*SIR2:HA* NEO upper	CCTGAAGCCATGATACTACGCTGCGAGGATGTGAGCTG
**UQ2704**	*SIR2:HA* NEO lower	TTATTCACCGATAGCTTGCGGGTTTATCTGTATTAACA
**UQ2705**	*SIR2:HA* 3’ upper	CAGCTCACATCCTCGCAGCGTAGTATCATGGCTTCAGG

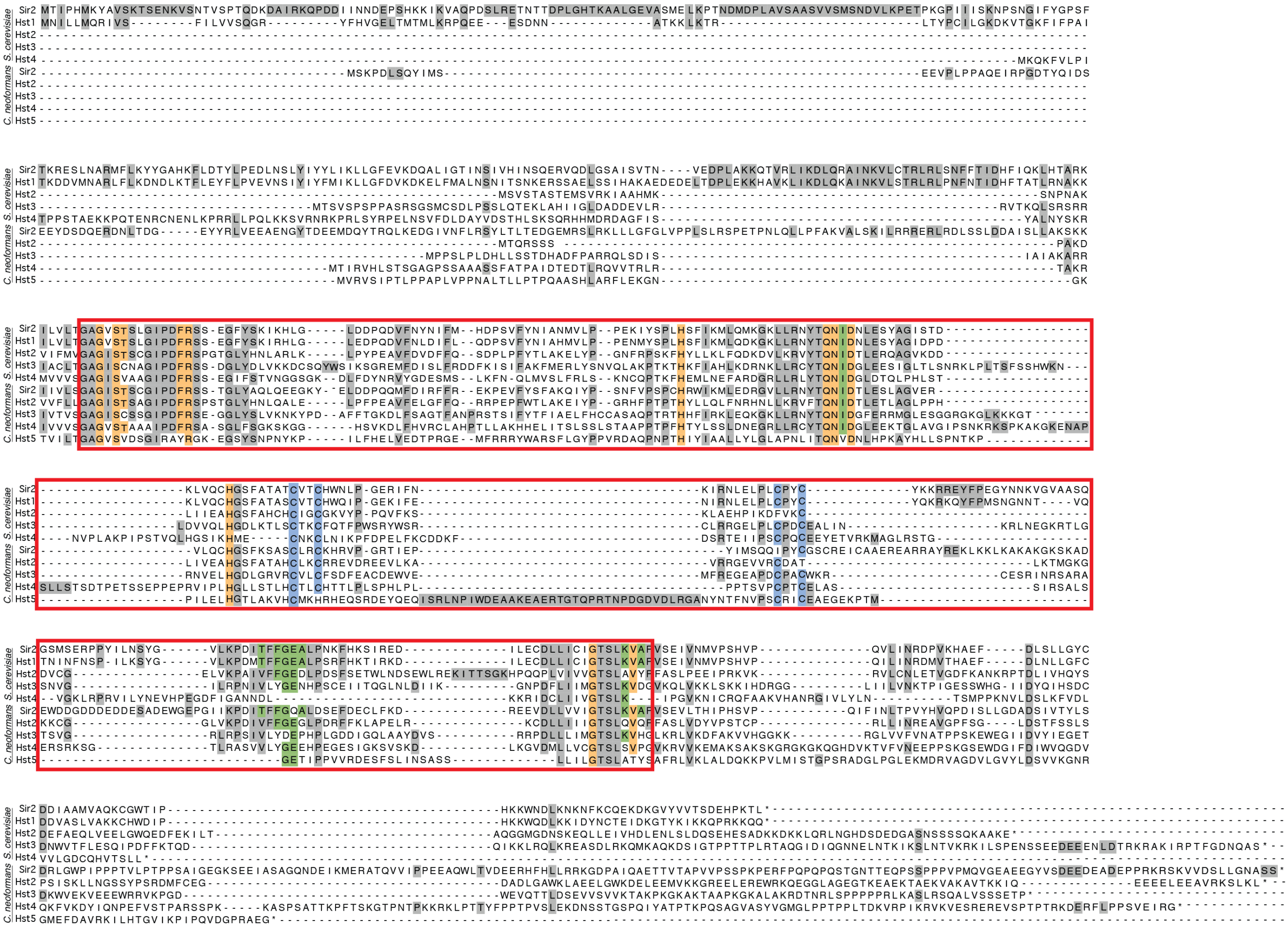

Supplementary Figure 1. *C. neoformans* and *S. cerevisiae* sirtuin protein alignment reveals divergent sequences but conserved regions for the NAD+, substrate, and zinc binding sites. Regions within the sirtuin domain (red) are more highly conserved than elsewhere in the proteins. Yellow indicates the amino acids involved in NAD+ binding, while blue and green are amino acids involved in zinc and substrate binding, respectively. Grey indicates amino acid residues that are identical in more than half of the proteins included in the analysis.

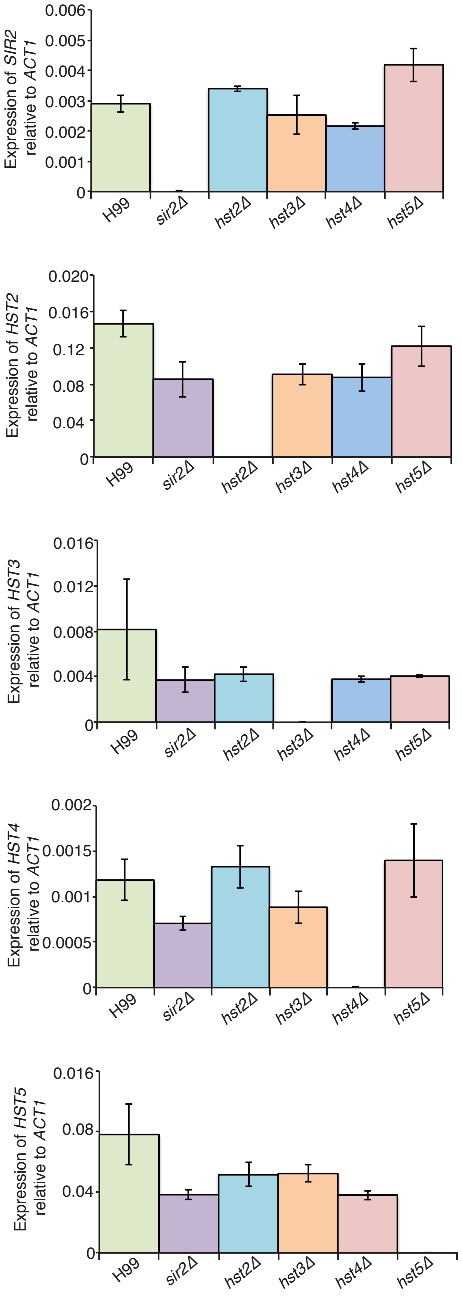

Supplementary Figure 2. Sirtuins do not regulate each other. qRT-PCR indicated that the siruin genes do not regulate each other, with the expression of the remaining sirtuin genes unchanged in each mutant Values show mean, error bars show.

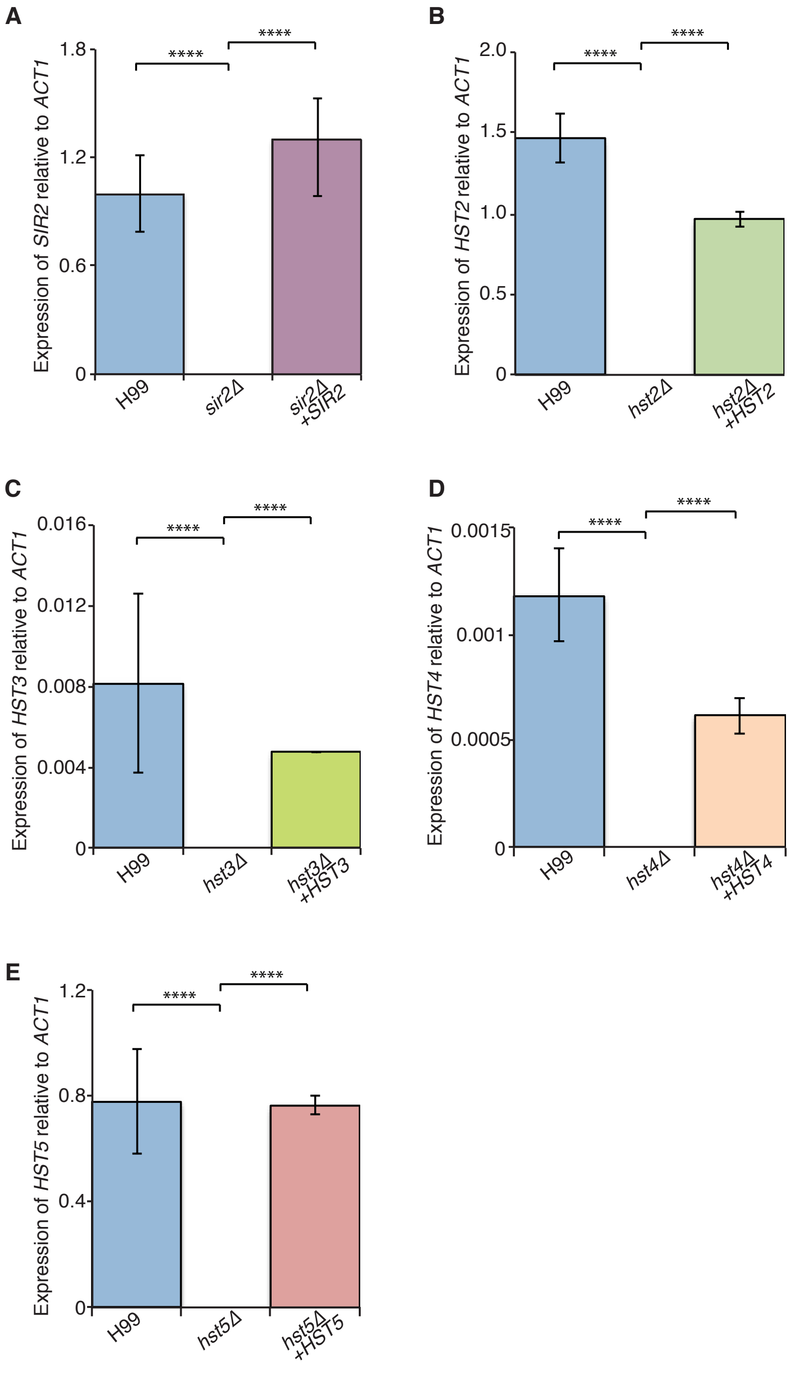

Supplementary Figure 3. Insertion of *SIR2* at the Safe Haven site in a *sir2*Δ strain has the same level of expression of *SIR2* compared with wild type. qRT-PCR indicated that expression of *SIR2* in the wildtype and complemented strain are not significantly different, while, as expected there is no expression of *SIR2* in the mutant strain. Values show mean, error bars show S.E.M, ***=P<0.001. **A**. SIR2, **B**. HST2, **C**. HST3, **D**. HST4, and **E**. HST5

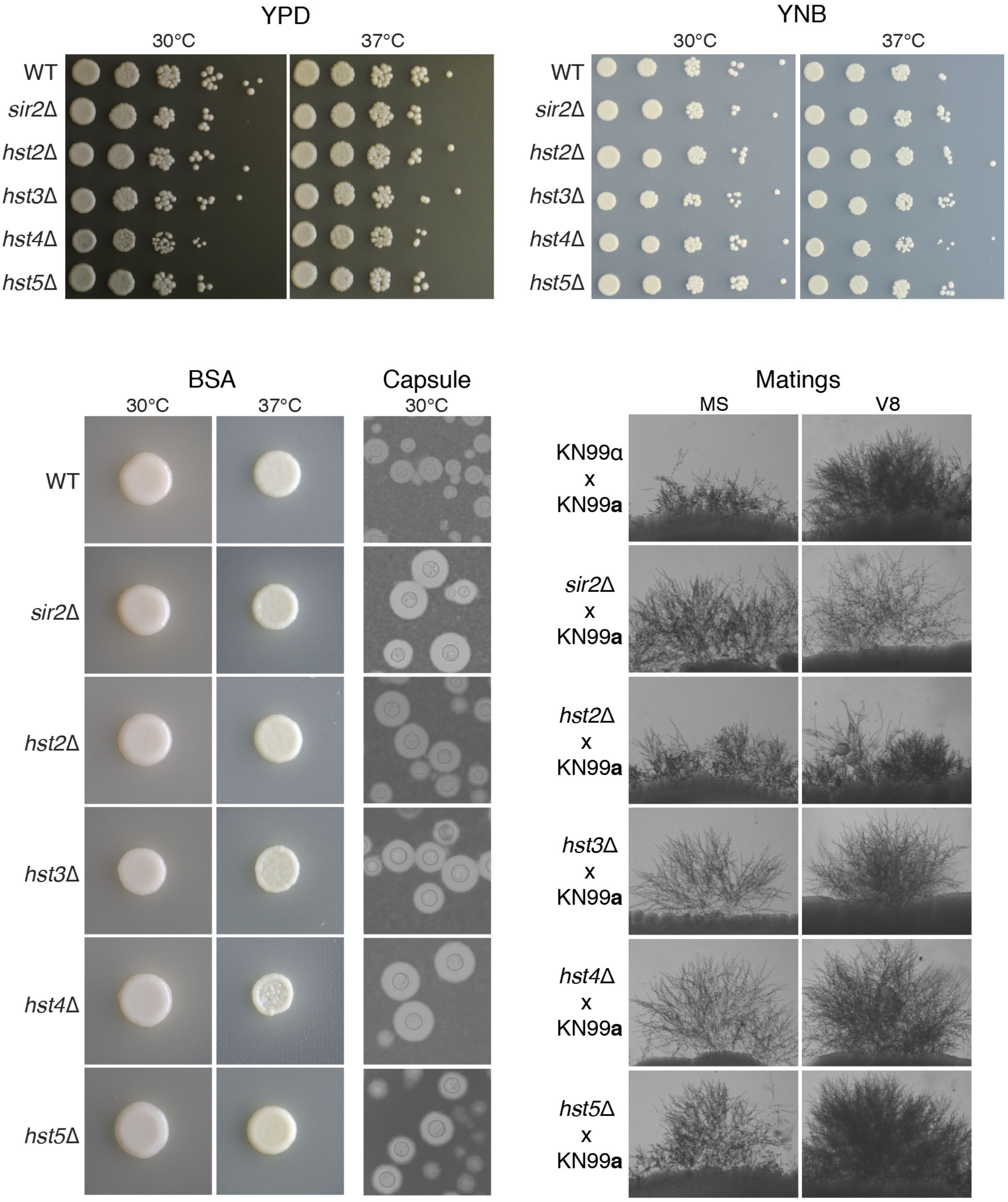

Supplementary Figure 4. The *C. neoformans* sirtuin mutants are indistinguishable from wild-type in certain *in vitro* virulence factor and mating assays. Wild-type phenotype was observed for all strains during growth on both rich (YPD) and minimal (YNB) media, following spot dilution assays on BSA agar, and when grown in capsule-inducing RPMI 1640 media with 10% FBS, 2% glucose for 24 hours. Mating assays were carried out on MS and V8 media, and kept in the dark at room temperature for two weeks.

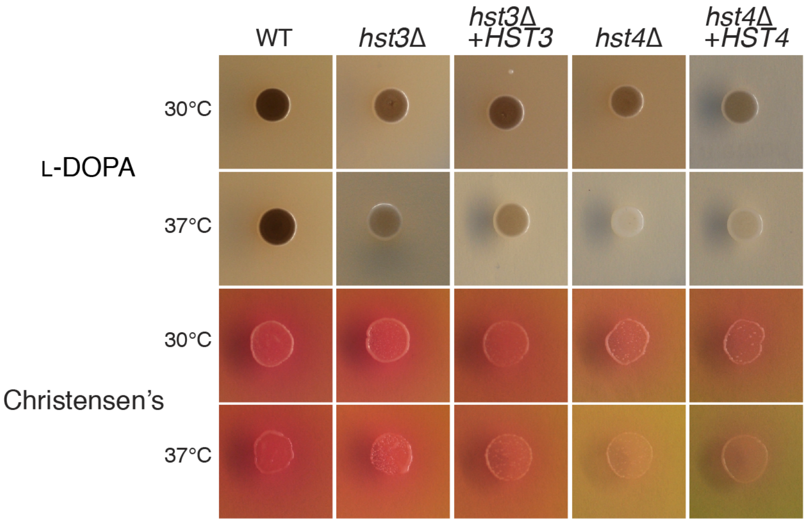

Supplementary Figure 5. Mutant phenotypes are not restored to wild-type after reintroduction of *HST3* and *HST4* into their respective mutants. Spot dilution assays on L-DOPA and Christensen's agar were used to determine the level of melanin and urease, respectively. Melanized strains appear brown-black, while urease production appears as a halo surrounding the colony. Plates were grown at both 30 and 37˚C.

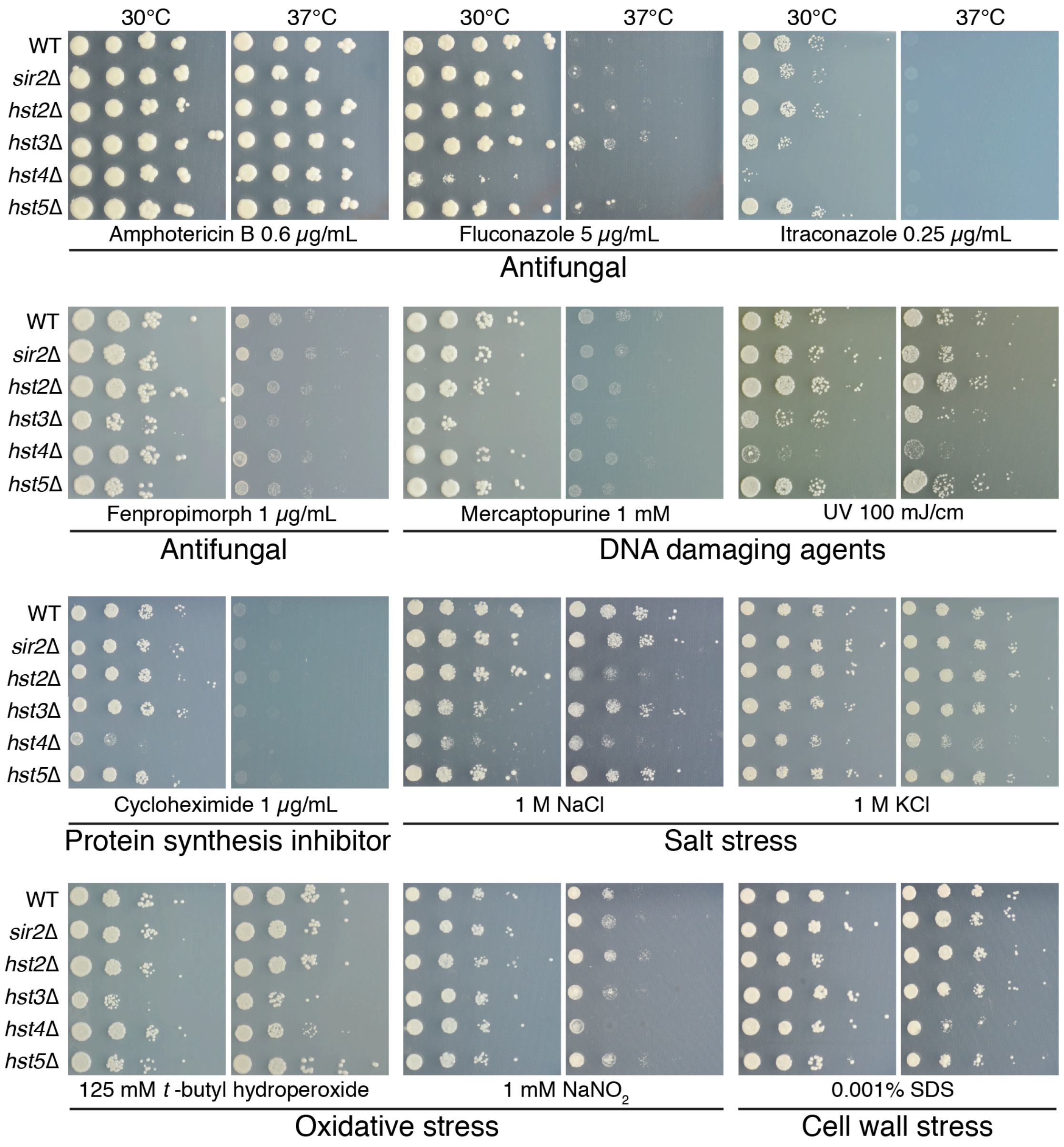

Supplementary Figure 6. Stress phenotypic assays reveal that *hst3*Δ and *hst4*Δ exhibit multiple *in vitro* phenotypes associated with stress conditions. 10-fold serial dilutions of indicated strains were spotted onto YNB media containing various stressors and incubated for 3 days at 30 and 37˚C.

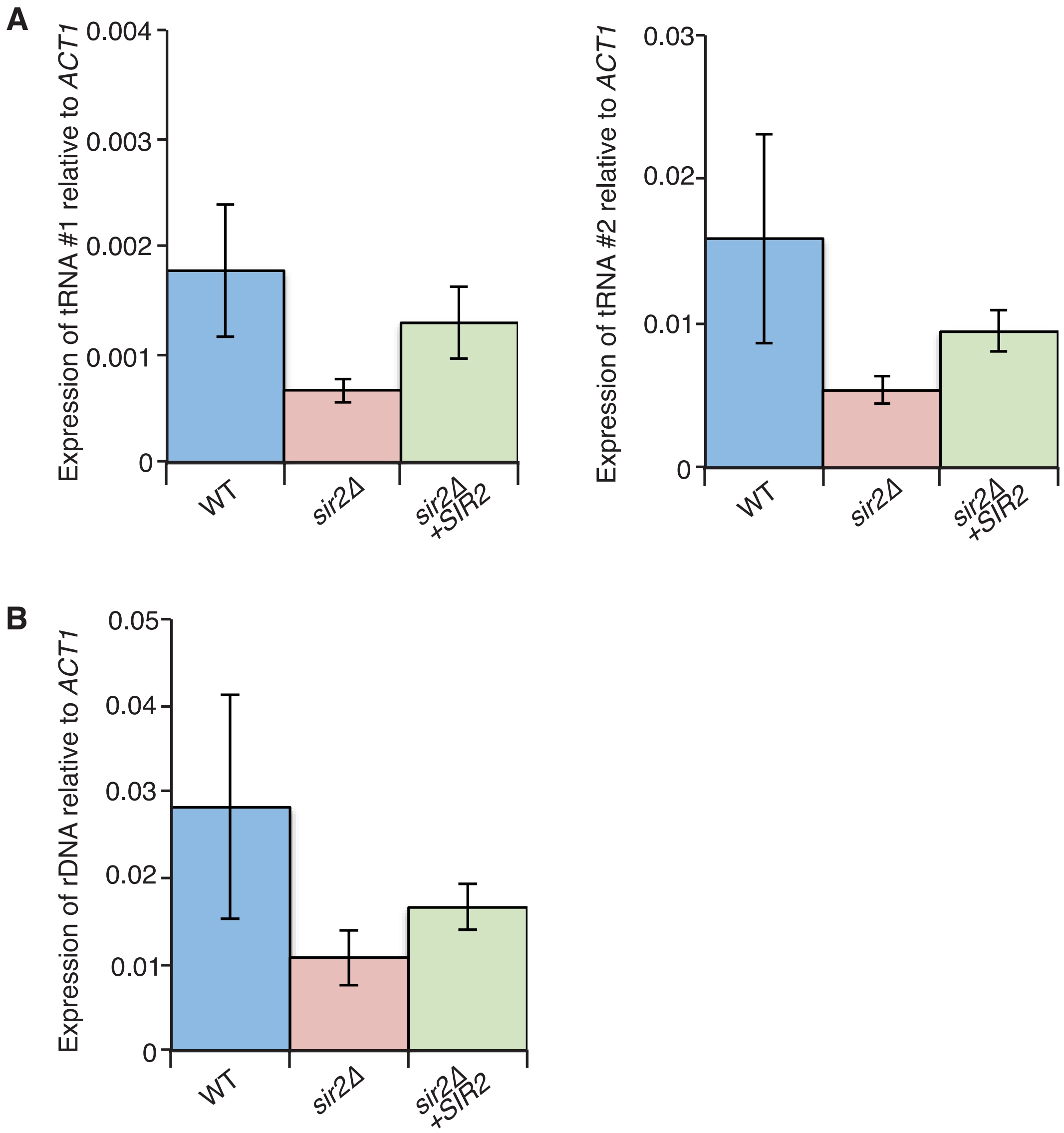

Supplementary Figure 7. Expression of two representative tRNAs and the rDNA array have the same level of expression of *SIR2* compared with wild type. Values show mean, error bars show S.E.M
